# YTHDF2 mediates the mRNA degradation of the tumor suppressors to induce AKT phosphorylation in N6-methyladenosine-dependent way in prostate cancer

**DOI:** 10.1186/s12943-020-01267-6

**Published:** 2020-10-29

**Authors:** Jiangfeng Li, Haiyun Xie, Yufan Ying, Hong Chen, Huaqing Yan, Liujia He, Mingjie Xu, Xin Xu, Zhen Liang, Ben Liu, Xiao Wang, Xiangyi Zheng, Liping Xie

**Affiliations:** Department of Urology, First Affiliated Hospital, School of Medicine, Zhejiang University, Hangzhou, Zhejiang China

**Keywords:** M^6^A, RNA methylation, YTHDF2, RNA degradation, Prostate cancer

## Abstract

**Background:**

N6-methyladenosine (m^6^A) is the most abundant modification in mRNA of humans. Emerging evidence has supported the fact that m^6^A is comprehensively involved in various diseases especially cancers. As a crucial reader, YTHDF2 usually mediates the degradation of m^6^A-modified mRNAs in m^6^A-dependent way. However, the function and mechanisms of m^6^A especially YTHDF2 in prostate cancer (PCa) still remain elusive.

**Methods:**

To investigate the functions and mechanisms of YTHDF2 in PCa, in vitro*,* in vivo biofunctional assays and epigenetics experiments were performed. Endogenous expression silencing of YTHDF2 and METTL3 was established with lentivirus-based shRNA technique. Colony formation, flow cytometry and trans-well assays were performed for cell function identifications. Subcutaneous xenografts and metastatic mice models were combined with in vivo imaging system to investigate the phenotypes when knocking down YTHDF2 and METTL3. m^6^A RNA immunoprecipitation (MeRIP) sequencing, mRNA sequencing, RIP-RT-qPCR and bioinformatics analysis were mainly used to screen and validate the direct common targets of YTHDF2 and METTL3. In addition, TCGA database was also used to analyze the expression pattern of YTHDF2, METTL3 and the common target LHPP in PCa, and their correlation with clinical prognosis.

**Results:**

The upregulated YTHDF2 and METTL3 in PCa predicted a worse overall survival rate. Knocking down YTHDF2 or METTL3 markedly inhibited the proliferation and migration of PCa in vivo and in vitro. LHPP and NKX3–1 were identified as the direct targets of both YTHDF2 and METTL3. YTHDF2 directly bound to the m^6^A modification sites of LHPP and NKX3–1 to mediate the mRNA degradation. Knock-down of YTHDF2 or METTL3 significantly induced the expression of LHPP and NKX3–1 at both mRNA and protein level with inhibited phosphorylated AKT. Overexpression of LHPP and NKX3–1 presented the consistent phenotypes and AKT phosphorylation inhibition with knock-down of YTHDF2 or METTL3. Phosphorylated AKT was consequently confirmed as the downstream of METTL3/YTHDF2/LHPP/NKX3–1 to induce tumor proliferation and migration.

**Conclusion:**

We propose a novel regulatory mechanism in which YTHDF2 mediates the mRNA degradation of the tumor suppressors LHPP and NKX3–1 in m^6^A-dependent way to regulate AKT phosphorylation-induced tumor progression in prostate cancer. We hope our findings may provide new concepts of PCa biology.

## Background

N6-methyladenosine (m^6^A) as the most abundant modification in messenger RNA (mRNA) of humans is a reversible process promoted by ‘writers’, inhibited by ‘erasers’ and functionally executed by readers [1, 2]. METTL3 and METTL14 are the core components that catalyze methylation sites supplemented with other regulatory proteins [3, 4]. Conversely, FTO and ALKBH5 eraser enzymes induce the demethylation process. The modified m^6^A sites are recognized and executed by variable readers to produce different functions or events. To date, YTH domain proteins (YTHDF1, YTHDF2, YTHDF3, YTHDC1 and YTHDC2) and HNRNP family proteins are primarily confirmed readers to regulate the pre-mRNA processing, translation and degradation processes [5–9]. Emerging evidence indicates that m^6^A is involved in various biological or physiological processes, and several disorders especially variable tumors [10–12]. It’s more convincing that the significant role of m^6^A modification is a new epigenetics concept known as “RNA epigenetics” or “epi-transcriptomics” to explain traditional problems in a novel way.

Prostate cancer (PCa) is the most diagnosed cancer among men in 105 countries, especially in developed countries. However, the mechanisms of tumorigenicity and potential target therapies for PCa, especially for high-risk PCa and castration-resistant PCa (CRPC) are still challenging [13]. Thus, further investigation of the profound mechanisms involved in PCa progression is extremely urgent.

Recently, surging evidence has elucidated the crucial role of m^6^A in regulating several human cancers [14–16]. However, the m^6^A regulatory mechanism in PCa progression still remains elusive. In this study, we primarily focused on the m^6^A modifications especially the reader YTHDF2, in regulating PCa progression. As a main cytoplasm m^6^A reader, YTHDF2 has been reported to degrade the m^6^A-modified mRNAs by binding to m^6^A sites to promote the tumor progression in several cancers [7, 17–19]. Moreover, this regulation mediated by YTHDF2 is dependent on m^6^A modifications catalyzed by METTL3-centred writers (m^6^A-dependent) [20]. Herein, we observed that YTHDF2 and METTL3 were significantly upregulated in PCa. Knocking down YTHDF2 or METTL3 inhibited the proliferation and migration by reducing AKT phosphorylation in vivo and in vitro. Screening by m^6^A RNA immunoprecipitation (MeRIP) sequencing (MeRIP-seq), RNA immunoprecipitation (RIP) RT-qPCR and online database analysis in PCa suggested that LHPP and NKX3–1 were the direct targets of YTHDF2 and METTL3. In addition, AKT phosphorylation was inhibited by overexpression of LHPP and NKX3–1. Thus, we proposed a novel regulatory mechanism that YTHDF2 mediates the mRNA degradation of tumor suppressors LHPP and NKX3–1 and indirectly induces AKT phosphorylation to promote PCa progression in m^6^A-dependent way. We expect that this study might provide a new regulatory concept of m^6^A in PCa and shed light for future biomarker diagnosis or targeted therapy of PCa.

## Materials and methods

### Cell lines and cell culture

The human normal prostate epithelial cell line RWPE-1 and PCa cell lines DU-145, PC-3, 22RV1 and VCaP were purchased from the Cell Bank of Type Culture Collection of Chinese Academy of Sciences (Shanghai, China), which were verified by short tandem repeat (STR) DNA profiling analysis. The RWPE-1 cell line was cultured in K-SFM medium (Gibco, 10,744–019) according to the product manual, DU-145 and PC-3 cell lines were cultured in MEM medium, 22RV1 cell line was cultured in RPMI-1640 (Glutamax and Sodium Pyruvate added), and VCaP cell line was cultured in DMEM (Glutamax and Sodium Pyruvate added), which were all supplemented with 10% heat-inactivated FBS and at 37 °C under a humidified atmosphere of 5% CO_2_.

### Reagents and transfection

Overexpression plasmids pYTHDF2, pLHPP and the control pNull (empty vector) were obtained from Genechem (Shanghai, China). FuGENE HD Transfection Reagent (Promega, Madison, USA) was used to transfect the overexpression plasmids according to the manufacturer’s protocol.

### Establishment of stable knock-down cell lines

We used the lentivirus-based shRNA vector GV344 (hU6-MCS-Ubiquitin-firefly_Luciferase-IRES-puromycin) obtained from Genechem (Shanghai, China) to infect the PC-3 and DU-145 cell lines, and finally screened out the stable knock-down cell lines with puromycin (2 μg/ml final concentration). All the associated sequences of shRNAs are listed as supplementary Table 1.

### Immunohistochemistry (IHC) staining

The tumors and metastases isolated from mice were sent for further IHC staining, which was performed as previously described [21, 22]. The antibodies used are listed as follows: anti-YTHDF2 (24744–1-AP, Proteintech), anti-METTL3 (ab195352, Abcam), anti-LHPP (15759–1-AP, Proteintech), and anti-NKX3–1 (sc-393,190, Santa Cruze Biotechnology).

### RNA m^6^A dot-blot assay

The RNA m^6^A dot-blot assay was used to evaluate the total RNA m^6^A levels. The specific procedures were performed according to previous study [23].

### MeRIP-seq and data analysis

Intact mRNA was isolated from total RNA samples using Arraystar Seq-StarTM poly(A) mRNA Isolation Kit, and then chemically fragmented (fragmentation buffer: 10 mM Zn2+,10 mM Tris-HCl, pH 7.0) to 100 nt in length. MeRIP was performed to enrich m^6^A methylated mRNA with m^6^A antibody (Abcam, ab208577). KAPA Stranded mRNA-seq Kit (Illumina, KK8421) was used for the library preparation of both m^6^A enriched RNA and input mRNAs and the quality control of the completed libraries was performed with Agilent 2100 Bioanalyzer. Then the libraries were sequenced on Illumina HiSeq 4000 system which was conducted by KangChen Bio-tech, Shanghai, China. With respect to the data analysis, sequencing reads were aligned to genome reference sequences using HISAT2 software (v2.1.0). The MeRIP enriched regions (peaks) were visualized by Integrative Genomics Viewer (IGV).

### MeRIP-RT-qPCR

All the specific manipulations were performed according to the protocol of Magna MeRIP™ m^6^A Kit (Catalog No. 17–10,499, Merck Millipore). The primers for RT-qPCR was listed in supplementary Table 2.

### RNA immunoprecipitation

All the specific manipulations were performed according to the protocol of EZ-Magna RIP™ RNA-Binding Protein Immunoprecipitation Kit (Cat. # 17–701, Merck Millipore). Briefly, 2.0 × 10^7^ cells (DU-145 and PC-3 cell lines) were lysed with lysis buffer and then processed for IP (5 μg of anti-YTHDF2 antibody 24,744–1-AP, Proteintech) overnight. The primers for RT-qPCR are listed in supplementary Table 2.

### RNA isolation and quantitative RT-PCR (RT-qPCR)

The specific procedures were performed as previously described [21]. The primers used in this study are listed in supplementary Table 2.

### Western blot assay

The specific procedures were conducted according to our previous study [22]. The primary immunoblotting antibodies used in this study were as follows: anti-GAPDH (10494–1-AP, Proteintech), anti-E-cadherin (20874–1-AP, Proteintech), anti-N-cadherin (66219–1-Ig), anti-PARP1 (13371–1-AP, Proteintech), anti-AKT (C67E7, Cell Signaling Technology), anti-pAKT (S473) (193H12, Cell Signaling Technology), and anti-pAKT(T308) (D25E6, Cell Signaling Technology). The antibodies of YTHDF2, LHPP, METTL3 and NKX3–1 was same with IHC. GAPDH was the internal reference.

### Colony formation assay

All specific manipulations were performed as previous studies [21].

### Flow cytometry assay

According to previous manipulations [22], treated cells were performed and analyzed with the BD LSRII Flow Cytometer System with FACSDiva Software (BD Bioscience, Franklin Lakes, USA).

### Wound-healing and trans-well assay

The specific procedures were consistent with a previous study [22]. Photographs were taken under phase-contrast microscopy (Olympus, Tokyo,Japan).

### Animal models and in vivo imaging

Approximately 2 × 10^6^ PCa cells (PC-3 shNC, shYTHDF2, shMETTL3 cell lines) per mouse suspended in 100 μl PBS were injected in the flank of male BALB/c nude mice (4 weeks old). During the 40-day observation, the tumor size (V = (width^2^ × length × 0.52)) was measured with vernier caliper. Approximately 1.5 × 10^6^ PCa cells suspended in 100 μl of PBS (PC-3 shNC, shYTHDF2, and shMETTL3 cell lines) per mouse were injected into the tail vein of male BALB/c nude mice (4 weeks old). The IVIS Spectrum animal imaging system (PerkinElmer) was used to evaluate the tumor growth (40 days) and whole metastasis conditions (4 weeks and 6 weeks) with 100 μl XenoLight D-luciferin Potassium Salt (15 mg/ml, Perkin Elmer) per mouse. Mice were anesthetized and then sacrificed for tumors and metastases which were sent for further organ-localized imaging as above, IHC staining and hematoxylin-eosin (H&E) staining. All animal studies and manipulations were performed in compliance with the institutional guidelines approved by the First Affiliated Hospital, School of Medicine, Zhejiang University.

### Databases used and KEGG pathway analysis

Several user-friendly databases or tools were utilized to download data, analyze or refer to in this study. TCGA database (https://portal.gdc.cancer.gov), Human Protein Atlas (https://www.proteinatlas.org/), Broad Institute Cancer Cell Line Encyclopedia (CCLE) (https://portals.broadinstitute.org/ccle), LinkedOmics online database (http://www.linkedomics.org/), DAVID (http://david.abcc.ncifcrf.gov/), SRAMP (http://www.cuilab.cn/sramp), Oncomine (https://www.oncomine.org/resource/login.html), and Venn diagram (http://bioinfogp.cnb.csic.es/tools/venny/index.html).

### Statistical analysis

The data were expressed as the mean ± S.D. Differences between groups were estimated using a student’s t-test, non-parametric test (Mann-Whitney test) or One-way ANOVA test with Bonferroni’s correction. Overall survival rate was calculated according to the Kaplan–Meier analysis and log-rank test. All analyses were performed using GraphPad prism 8.0 (La Jolla, CA, USA) or SPSS StatisticsV25 software (Armonk, NY, USA) and a two-tailed value of *P ≤ 0.05, **P ≤ 0.01, and ***P ≤ 0.001 were considered statistically significant.

## Results

### YTHDF2 and METTL3 are frequently upregulated in PCa tissues and cell lines

**Fig. 1:**
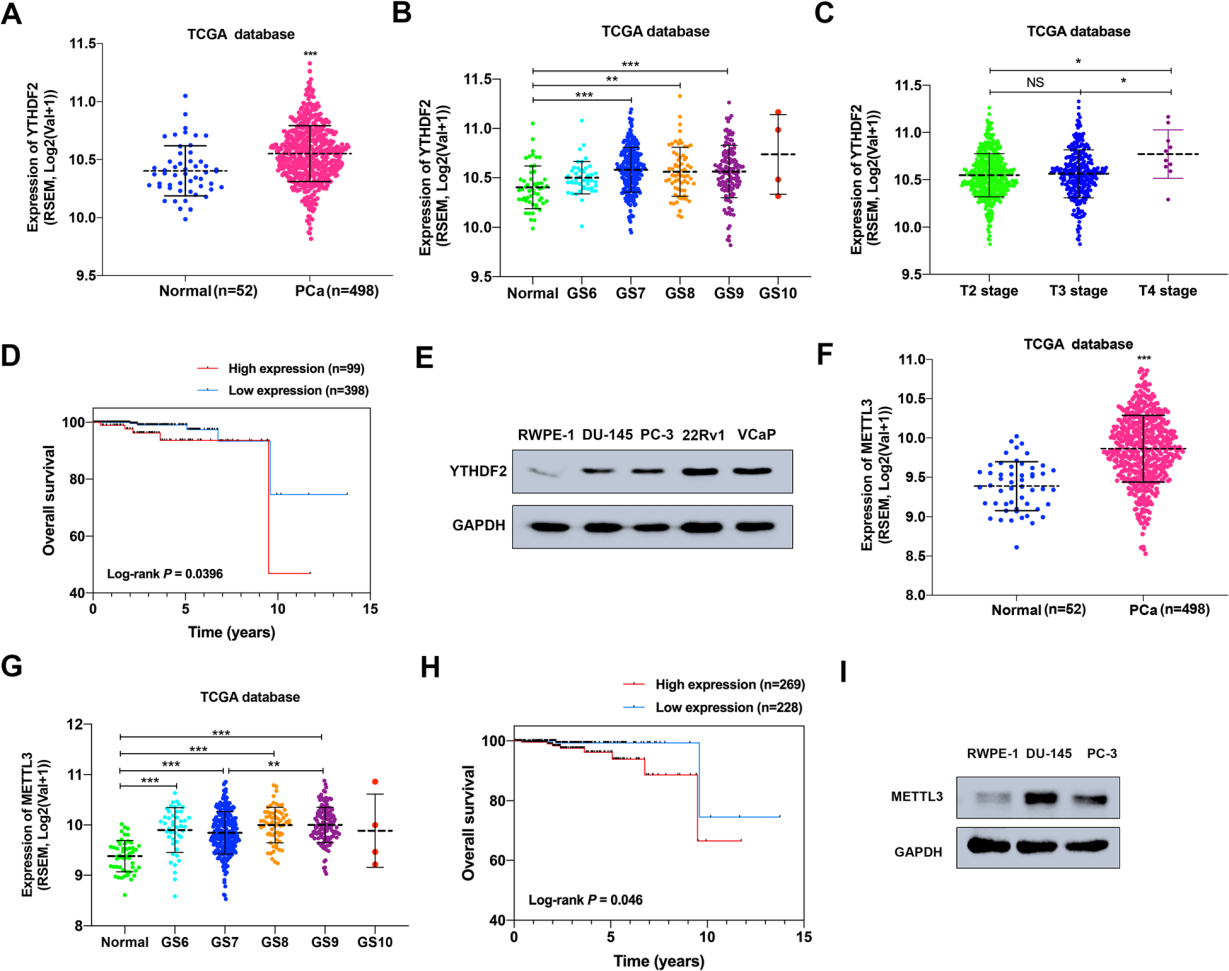
YTHDF2 and METTL3 are frequently upregulated in PCa. **a**-**c** The expression pattern of YTHDF2 in total, separate Gleason scores or different T stages were analyzed in 498 PCa tissues and 52 normal controls (TCGA database). Student’s t test was used for statistical analysis of two groups. One-way ANOVA test with Bonferroni’s correction was used for statistical analysis of more than two groups comparisons. **d** Kaplan–Meier curve was used to evaluate the overall survival rate in a cohort of 497 PCa patients according to the relative mRNA expression of YTHDF2. *P* value (*P* = 0.0396) was calculated with log-rank test. **e** The protein level of YTHDF2 in RWPE-1 and PCa cell lines analyzed by the western blot assay. GAPDH was the internal reference. **f**, **g** The expression pattern of METTL3 in total or in separate Gleason score in 498 PCa tissues (52 normal controls) were analyzed by TCGA database. Student’s t test was used for the statistical analysis of two groups comparison. One-way ANOVA test with Bonferroni’s correction was used for statistical analysis of more than two groups comparisons. **h** The survival probability of METTL3 in PCa patients was analyzed with Kaplan–Meier analysis and log-rank test in 497 PCa patients according to the relative expression of METTL3. **i** The protein level of METTL3 in RWPE-1 and PCa cell lines analyzed by the western blot assay. GAPDH was the internal reference. Error bars represent the SD obtained from at least three independent experiments; *P ≤ 0.05, **P ≤ 0.01, ***P ≤ 0.001

To investigate the expression pattern of YTHDF2 and METTL3 in PCa, TCGA and Oncomine databases were utilized. Upregulation of YTHDF2 was observed in TCGA PCa tissues (*n* = 498) compared with the normal controls (*n* = 52) (*P* < 0.001) and in Grasso Prostate cohort (Oncomine database) (Fig. 1a, supplementary figure 1A), and the expression of YTHDF2 tended to increase with the growing Gleason scores (Fig. 1b). Furthermore, higher PCa T stages presented the higher YTHDF2 level (Fig. 1c). The survival probability was also analyzed in 497 patients, and the result revealed that a poorer survival rate was along with a higher expression level of YTHDF2 in PCa patients (*P* = 0.0396) (Fig. 1d). In addition, western blot assay and CCLE data also confirmed the higher expression of YTHDF2 in PCa cell lines (DU-145, PC-3, 22RV1, VCaP) than the normal prostate epithelial cell line (RWPE-1) (Fig. 1e, supplementary figure 2A). As the crucial m^6^A writer, METTL3 was also upregulated in TCGA PCa tissues (*n* = 498) compared to the normal controls (*n* = 52) (*P* < 0.001) (Fig. 1f) and in several cohorts from Oncomine (supplementary figure 1B-F), and there is an increasing tendency of METTL3 expression was associated with higher Gleason scores (Fig. 1g). Survival probability in 497 patients from TCGA was analyzed and suggested that patients with higher METTL3 expression had a poorer survival rate (*P* = 0.046) (Fig. 1h). The protein level of METTL3 in DU-145 and PC-3 cell lines were also higher than RWPE-1 cell line identified by western blot (Fig. 1i). The expression pattern and clinical value of other m^6^A writers and erasers were also investigated by analyzing TCGA, CCLE data and western blot assay (supplementary figure 2B-F and 3A-H), however, no significant results were found. The above results indicate that upregulated YTHDF2 and METTL3 may be involved in PCa progression.

### Knock-down of YTHDF2 significantly inhibits PCa progression in vitro

**Fig. 2:**
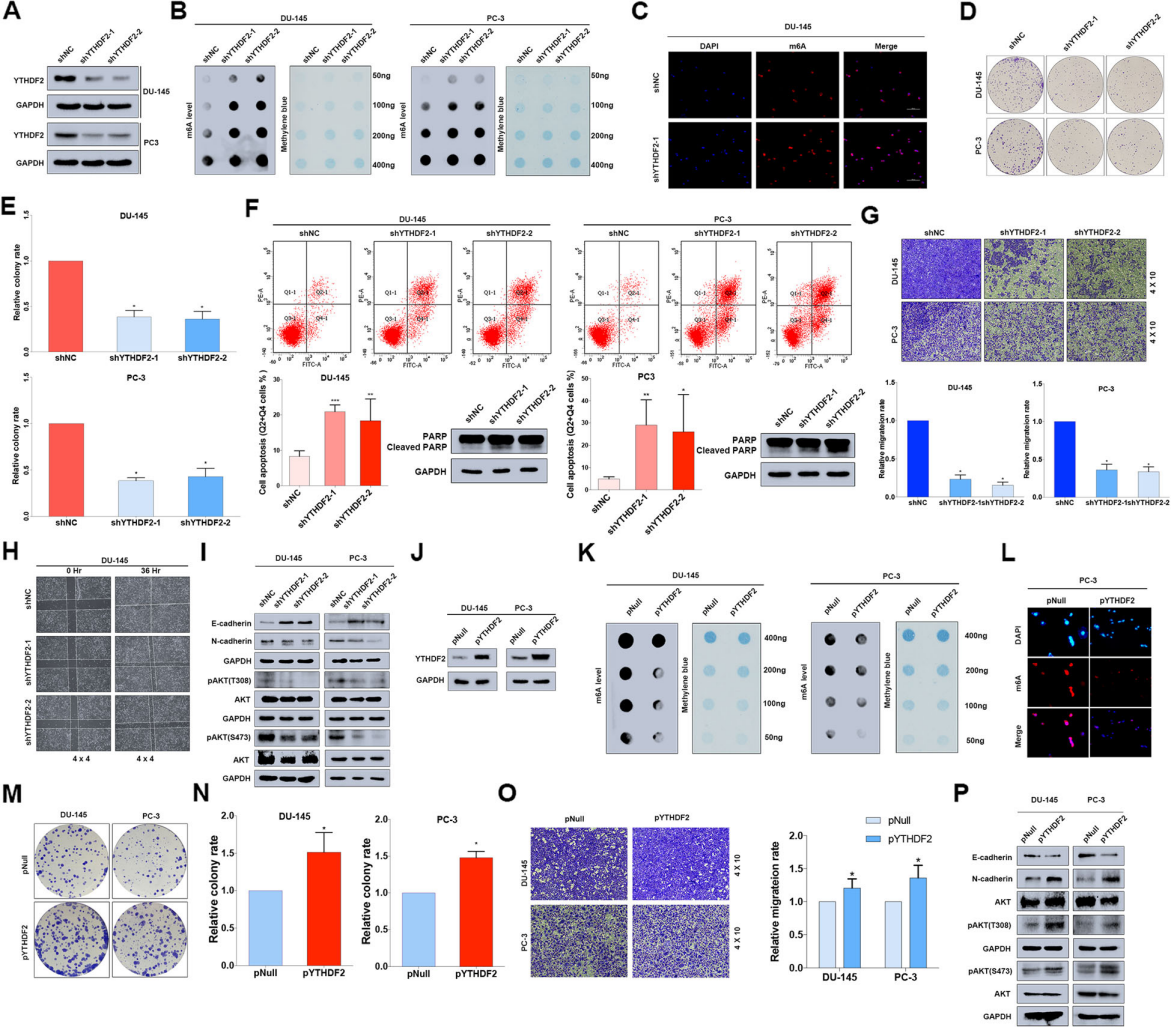
Knock-down of YTHDF2 inhibits the tumor progression of PCa in vitro. **a** The knock-down efficiency of YTHDF2 shRNAs (shYTHDF2–1 and shYTHDF2–2) with lentivirus constructs in DU-145 and PC-3 cell lines was confirmed by western blot. GAPDH was the internal reference. **b** m^6^A RNA dot-blot assay. The m^6^A level alterations at different total RNA concentrations (50 ng, 100 ng, 200 ng, 400 ng) in DU-145 and PC-3 cell lines were detected. Methylene blue staining was loading control. **c** The m^6^A levels detected by IF in DU-145 cell line after knocking down YTHDF2 (shYTHDF2–1), scale bar = 100 μm. **d** The proliferation ability after knocking down YTHDF2 was evaluated by colony formation assay (representative wells were presented) and (**e**) statistically analyzed by Mann-Whitney test. **f** Flow cytometry assay (representative images were presented) and western blot assay were used to confirm the apoptosis analysis induced by knock-down of YTHDF2. GAPDH was the internal reference. Student’s t test was used for the statistical analysis. **g** and **h** Trans-well assay and wound-healing assay (representative wells were presented) were assessed for the cell migration in YTHDF2 knock-down cell lines. Mann-Whitney test was used for the statistical analysis. **i** Western blot assay was used to detect the alterations of EMT-associated proteins and AKT phosphorylation level in YTHDF2 knock-down PCa cell lines. GAPDH was the internal reference. **j** The overexpression efficiency of pYTHDF2 plasmid (transient transfection with FuGENE HD Transfection Reagent, 0.5 μg/ml) compared with control pNull in DU-145 and PC-3 cell lines was detected by western blot assay. GAPDH was the internal reference. **k** and **l** m^6^A RNA dot-blot assay and IF assay were used to determine the m^6^A levels after overexpression of YTHDF2. Methylene blue staining was loading control. **m** Proliferation ability was evaluated by colony formation assay (representative wells were presented) in YTHDF2-overexpressed cell lines, and (**n**) statistically analyzed with Mann-Whitney test. **o** Trans-well assay (representative wells were presented) was used to detect the cell migration. Mann-Whitney test was used for the statistical analysis. **p** The alterations of EMT-associated proteins and AKT phosphorylation level were all detected by western blot assay after overexpression of YTHDF2 in DU-145 and PC-3 cell lines. GAPDH was the internal reference. Error bars represent the SD obtained from at least three independent experiments; *P ≤ 0.05, **P ≤ 0.01, ***P ≤ 0.001

Through lentivirus-based small hairpin RNAs (shRNAs) technique, the endogenous expression of YTHDF2 was effectively knocked down in DU-145 and PC-3 cell lines (Fig. 2a). As an important m^6^A modification reader, YTHDF2 was previously confirmed to upregulate m^6^A levels by inducing targeted mRNA decay [7, 24]. To investigate the alterations of m^6^A levels, RNA m^6^A dot-blot assay was used, and the results revealed an upregulation of total m^6^A levels in YTHDF2 knock-down cell lines when RNA concentrations varied (50 ng, 100 ng, 200 ng and 400 ng) (Fig. 2b), which was also consistently corroborated by cell immunofluorescence of m^6^A (Fig. 2c).

In terms of the potential function of YTHDF2 in PCa cell lines, we performed the cell proliferation and migration associated assays. The colony formation assay revealed that knock-down of YTHDF2 reduced the colony formation rate in DU-145 and PC-3 cell lines (Fig. 2d and e). A further flow cytometry assay of two cell lines suggested that apoptosis induced by YTHDF2 knock-down may be responsible for the inhibition of proliferation, and the induction of cleaved PARP1 was observed to confirm the apoptosis result (Fig. 2f). The cell migration rate was also obviously abrogated by knock-down of YTHDF2 through trans-well assay and wound-healing assay (Fig. 2g and h). The western blot assay shown that epithelial–mesenchymal transition (EMT)-associated proteins and pAKT (S473 and T308) were inhibited (Fig. 2i), indicating that YTHDF2 is involved in AKT phosphorylation to regulate PCa progression. Conversely, forced expression with transfection of the overexpression plasmid (pYTHDF2) (Fig. 2j) reduced total m^6^A levels of DU-145 and PC-3 cell lines at different RNA concentrations (50 ng, 100 ng, 200 ng and 400 ng) (Fig. 2k), and the results were also corroborated by m^6^A immunofluorescence in PC-3 cell line (Fig. 2l). Colony formation assay and trans-well assay all suggested that overexpression of YTHDF2 promoted the cell proliferation and migration (Fig. 2m, n and o). In addition, a further western blot assay also validated that EMT progression and AKT phosphorylation were induced by YTHDF2 (Fig. 2p). Downstream GSK-3β/SNAIL signal pathway was detected involved in YTHDF2 mediated EMT progression (Supplementary figure 4). Both rescue experiment with overexpressing wildtype YTHDF2 and 3’UTR targeting siRNA of YTHDF2 transfection were performed to confirm the knock-down effect of YTHDF2, which revealed the consistent results (supplementary figure 5 and 6). Considering the above results, we suspected that upregulated oncogene YTHDF2 was involved in PCa proliferation and migration by regulating m^6^A levels and phosphorylated AKT signal pathway.

### Knock-down of YTHDF2 inhibits tumor growth and metastasis in vivo

**Fig. 3:**
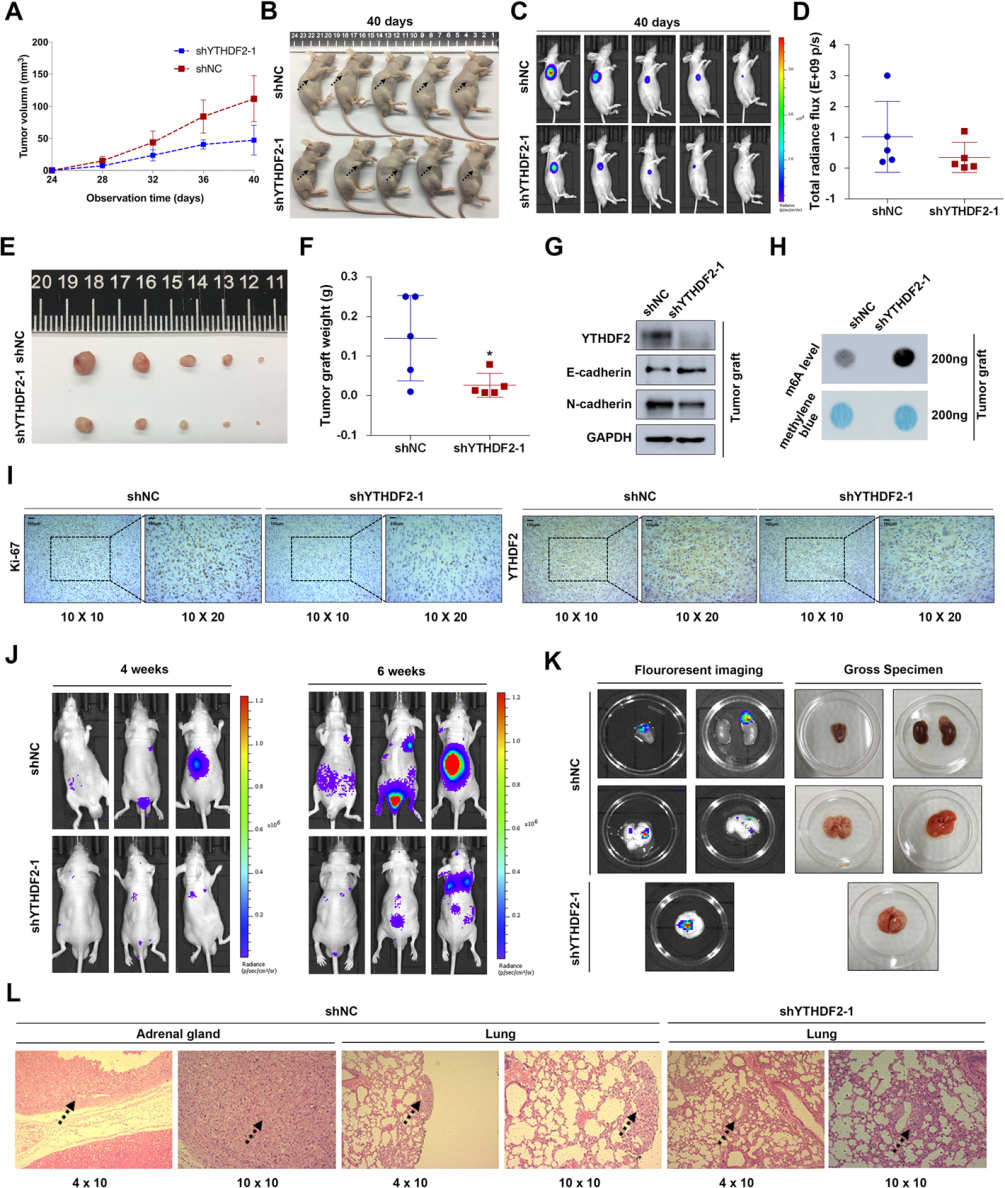
Knock-down of YTHDF2 inhibits tumor growth and metastasis in vivo. **a-i** Subcutaneous tumor model (BALB/c nude mice). **a** The tumor growth curve of xenografts was plotted in shNC and shYTHDF2 group (*n* = 5 each group) by measuring the tumor size (width^2^ × length × 0.52) with vernier caliper each 4 days. **b** The subcutaneous tumor models were observed at 40 days in two different groups (blank arrows indicated tumor xenografts). **c** and **d** The luciferase activities (radiance values) of subcutaneous tumor xenografts were measured at 40 days by in vivo imaging system in two groups. The values of above groups were analyzed with student’s t test. **e** The BALB/c nude mice were sacrificed for the xenografts, and the size was measured by the beside ruler. **f** The anatomized subcutaneous tumor xenografts were weighed and analyzed with student’s t test between two groups. **g** The EMT associated proteins and YTHDF2 protein extracted from anatomized tumor xenografts were analyzed by western blot assay. GAPDH was the internal reference. **h** Total RNA was extracted from the tumor xenografts, and m^6^A levels were determined by m^6^A RNA dot-blot assay. Methylene blue staining was loading control. **i** Representative IHC staining micrographs of Ki-67, YTHDF2 in tumor xenografts were conducted. Scale bar = 100 μm. **j**-**l** Metastatic model (BALB/c nude mice). **j** The BALB/c nude mice injected with cells (1.5 × 10^6^ per mouse) via tail vein were in vivo imaged at 4th weeks and 6th weeks to evaluate the whole metastasis. **k** The mice were sacrificed for the metastatic organs which were further in vivo imaged to reconfirm the metastasis. The representative photographs and corresponding gross specimens (right panel) were presented. **l** Representative H&E staining of metastatic organs (adrenal gland and lung) were performed to identify the metastasis loci. Error bars represent the SD obtained from at least three independent experiments; *P ≤ 0.05, **P ≤ 0.01, ***P ≤ 0.001

The PC-3 cell line (shYTHDF2) established with a lentivirus-based shRNA technique was stably knocked down for YTHDF2 and stably expressed luciferase. It was used to establish animal xenograft model. During the observation of flank xenograft in BALB/c nude mice for 40 days, we found that knocking down YTHDF2 resulted in a dramatic retardation of tumor growth (Fig. 3a and b), which was consistent with the radiance value tested by an in vivo imaging system (Fig. 3c and d). The nude mice were consequently sacrificed and the xenografts were anatomized and isolated (Fig. 3e). The tumor weights of two groups were significantly different (Fig. 3f). The xenografts were lysed to extract the proteins and total RNA. The western blot assay indicated an obvious knock-down of YTHDF2 and a reversed EMT-associated proteins in the shYTHDF2 group (Fig. 3g). In addition, the total m^6^A levels were also detected in the total RNA of extracted xenograft, and the results revealed that knocking down YTHDF2 markedly upregulated the total m^6^A levels in vivo (Fig. 3h). To validate the expression of YTHDF2-targeted genes and Ki-67 in xenograft tissues, IHC staining was used and revealed that the protein levels of Ki-67 and YTHDF2 were inhibited (Fig. 3i). The whole metastatic model was established and detected using an in vivo imaging system after injecting PC-3 shYTHDF2 and shNC cells into tail vein of nude mice. A reduction of whole metastasis sites was observed in shYTHDF2 group compared to the shNC control group at the 4th week and 6th week after injections (Fig. 3j). The metastatic organs were anatomized and imaged using the in vivo imaging system to further confirm the metastasis (Fig. 3k). The isolated metastatic tissues were further made into slides and stained with H&E to validate and locate the metastasis (Fig. 3l). In summary, knocking down YTHDF2 dramatically inhibited the tumor growth and metastasis in vivo.

### Knock-down of METTL3 significantly inhibits PCa progression in vitro

**Fig. 4:**
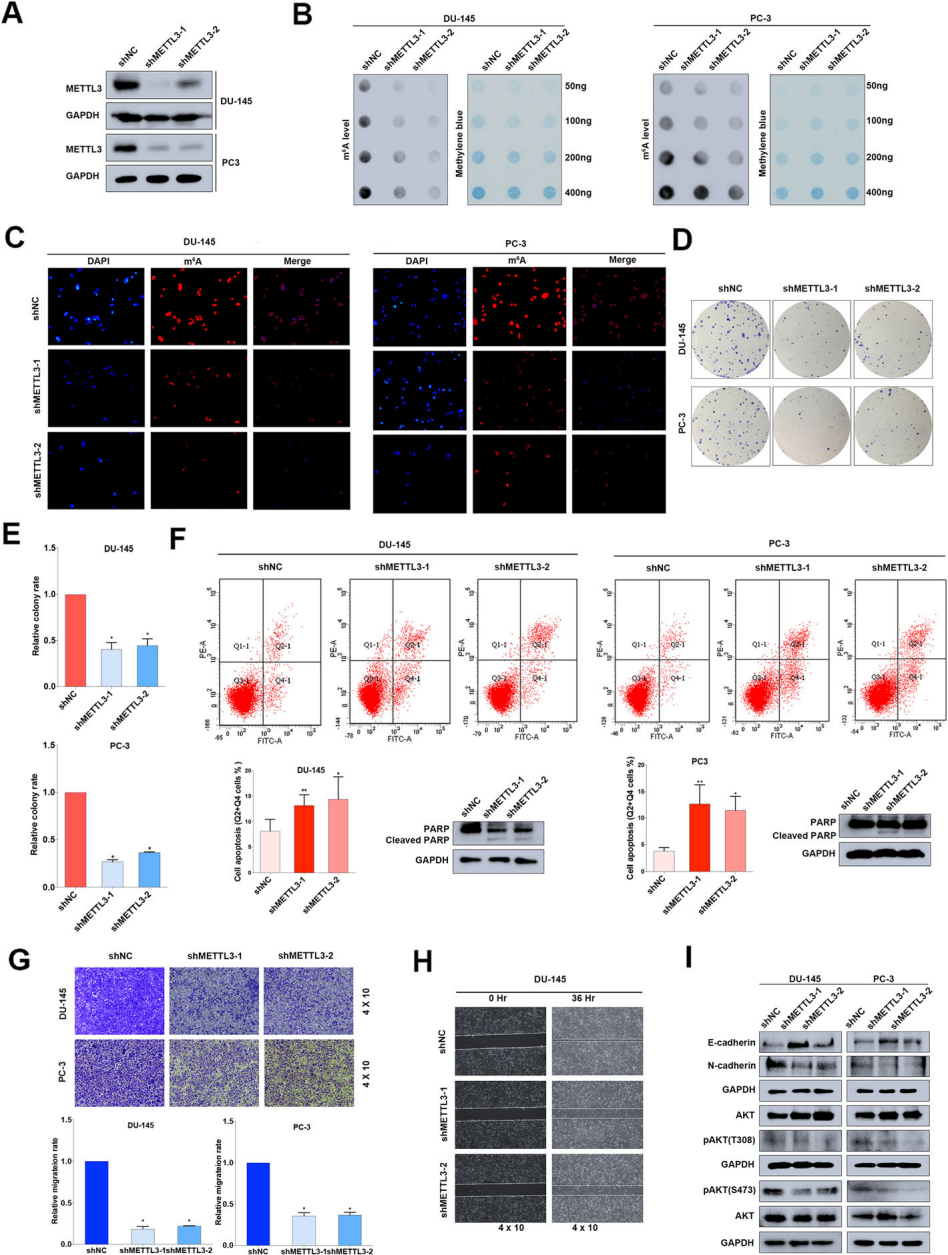
Knock-down of METTL3 inhibits PCa progression in vitro. **a** The knock-down efficiency of METTL3 shRNAs (shMETTL3–1, shMETTL3–2) with lentivirus constructs in DU-145 and PC-3 cell lines were detected by western blot assay. GAPDH was the internal reference. **b** and **c** m^6^A RNA dot-blot assay and IF were used to evaluate the m^6^A level alterations after knocking down METTL3. Methylene blue staining was loading control. **d** and **e** The proliferation ability was evaluated by colony formation assay after knocking down METTL3 (representative wells were presented) and statistically analyzed by Mann-Whitney test. **f** Flow cytometry assay and western blot assay were used to evaluate the apoptosis analysis induced by knock-down of METTL3. GAPDH was the internal reference. Student’s t test was used for the statistical analysis. **g** and **h** The trans-well assay and wound-healing assay (representative wells were presented) were used to determine the cell migration after knocking down METTL3. Mann-Whitney test was used for the statistical analysis. **i** The EMT-associated proteins and AKT phosphorylation were detected by western blot assay. GAPDH was the internal reference. Error bars represent the SD obtained from at least three independent experiments; *P ≤ 0.05, **P ≤ 0.01, ***P ≤ 0.001

METTL3 as the core writer, it’s frequently reported to mediate RNA metabolism cooperating with the downstream directly-executive reader YTHDF2 in various diseases especially in cancers. Considering the upregulation of METTL3 in PCa, in this section we focused on its specific role in PCa. A lentivirus-based shRNAs technique was utilized to efficiently knock down the endogenous expression of METTL3 in DU-145 and PC-3 cell lines (Fig. 4a). The RNA m^6^A dot-blot assay suggested that the total m^6^A levels were significantly reduced in METTL3 knock-down cell lines at different RNA concentrations (50 ng, 100 ng, 200 ng and 400 ng) (Fig. 4b, supplementary figure 7A-C), which was additionally validated by the downregulation of m^6^A immunofluorescence after knocking down METTL3 (Fig. 4c). In terms of cell function assays, knock-down of METTL3 significantly inhibited the colony formation rate in DU-145 and PC-3 cell lines (Fig. 4d and e). In addition, flow cytometry suggested that apoptosis could be triggered by knocking down METTL3, which was confirmed by upregulated cleaved PARP1 (Fig. 4f). The trans-well assay and wound-healing assay were also used to investigate the migration ability, and the results indicated that knock-down of METTL3 significantly repressed the migration rate in DU-145 and PC-3 cell lines (Fig. 4g and h). EMT-associated proteins were consistently reversed, and AKT phosphorylation was also inhibited by silencing METTL3 (Fig. 4i). Both Rescue experiment with overexpressing wildtype METTL3 and 5’UTR targeting siRNA of METTL3 transfection were performed to confirm the knock-down effect of METTL3, which revealed the consistent results (supplementary figure 5 and 6). According to the above results, we found that both METTL3 and YTHDF2 could regulate AKT phosphorylation. To conclude, METTL3 is involved in PCa progression by producing m^6^A modified sites of target mRNAs which were recognized by YTHDF2.

### Knock-down of METTL3 inhibits the tumor growth and metastasis in vivo

**Fig. 5:**
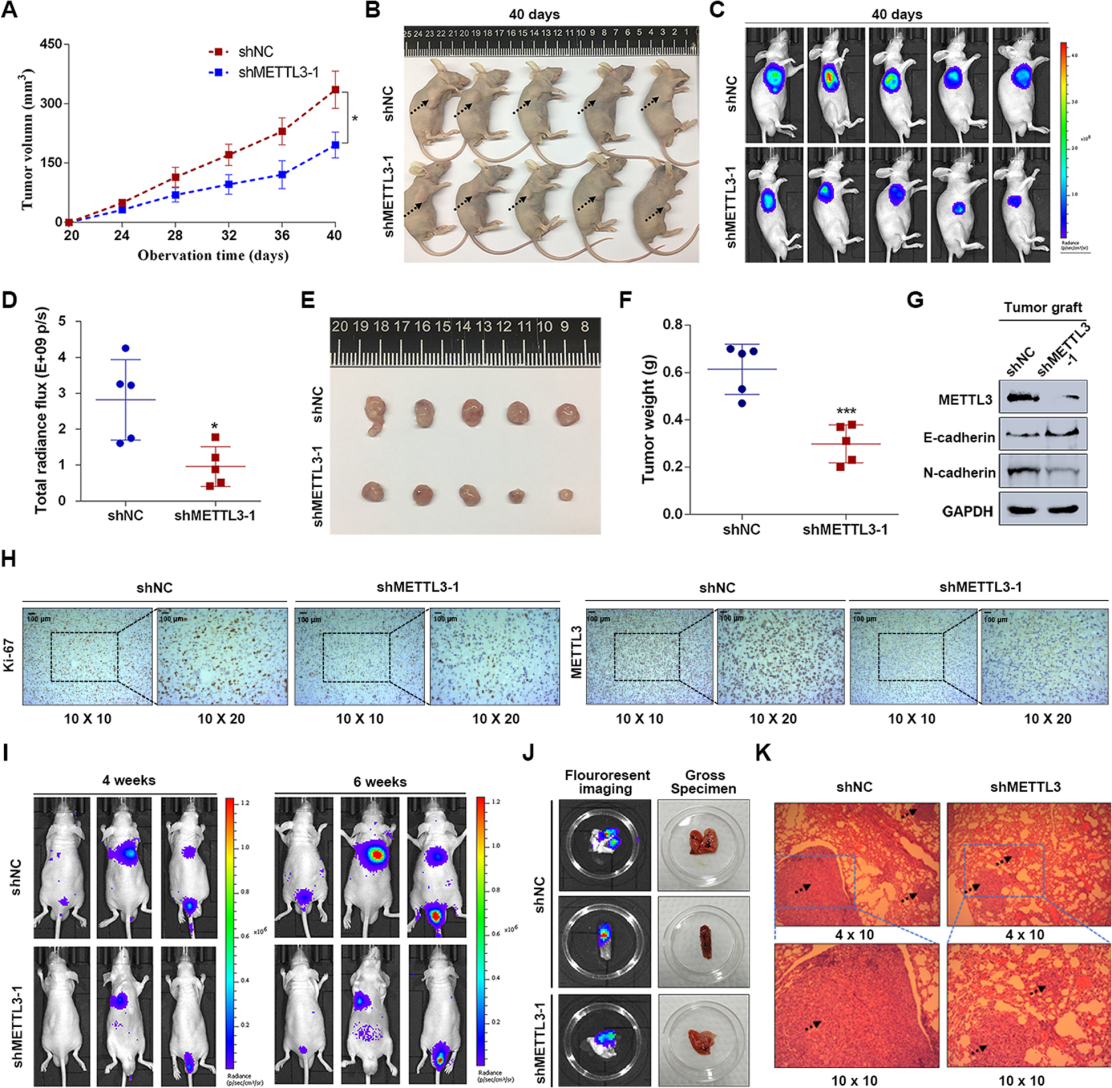
Knock-down of METTL3 inhibits tumor growth and metastasis in vivo. **a**-**h** Subcutaneous tumor model (BALB/c nude mice). **a** The tumor growth curve of xenografts was plotted in shNC and shMETTL3 group (*n* = 5 each group) by measuring the tumor size (width^2^ × length × 0.52) with vernier caliper. The tumor size at the endpoint in above group was analyzed with student’s t test. **b** The subcutaneous tumor models were observed at 40 days in two different groups (blank arrows indicated tumor xenografts). **c** and **d** The luciferase activities (radiance values) of subcutaneous tumor xenografts were measured by in vivo imaging system in two groups. The values of above were analyzed with student’s t test. **e** The BALB/c nude mice were sacrificed for the xenografts, and the size was measured by the beside ruler. **f** The anatomized subcutaneous tumor xenografts were weighed and analyzed with student’s t test. **g** The EMT associated proteins and METTL3 protein extracted from anatomized tumor xenografts were analyzed by western blot assay. GAPDH was the internal reference. **h** Representative IHC staining micrographs of Ki-67, METTL3 in tumor xenografts were conducted. Scale bar = 100 μm. **i**-**k** Metastatic model (BALB/c nude mice). **i** The BALB/c nude mice injected with cells (1.5 × 10^6^ per mouse) via tail vein were in vivo imaged at 4th weeks and 6th weeks to evaluate the whole metastasis. **j** The mice were sacrificed for the metastatic organs which were further in vivo imaged to reconfirm the metastasis. The representative photographs and corresponding gross specimens (right panel) were presented. **k** H&E staining of several metastatic organs (lung) were performed to identify the metastasis loci. Error bars represent the SD obtained from at least three independent experiments; *P ≤ 0.05, **P ≤ 0.01, ***P ≤ 0.001

To investigate the role of METTL3 in vivo, we established a METTL3 knock-down and stably expressing luciferase PC-3 cell line (shMETTL3) and control shNC cell line. The subcutaneous tumor xenograft model results indicated that knocking down METTL3 induced an obvious retardation of tumor growth (Fig. 5a and b), which was further corroborated by the reduced radiance value measured by the in vivo imaging system after 40 days (Fig. 5c and d). The nude mice were sacrificed at 40 days, and the flank xenografts were anatomized and weighed, which indicated an obvious reduction of volume and weight in the shMETTL3 group (Fig. 5e and f). The proteins extracted from the anatomized xenografts were analyzed by western blot assay, and the results indicated that METTL3 was downregulated and EMT associated proteins were reversed in shMETTL3 group (Fig. 5g). Furthermore, IHC staining of Ki-67, METTL3was also performed in these tissues (Fig. 5h), and the results were consistent with knock-down of YTHDF2. Tail vein injection metastatic model was performed to evaluate the whole metastasis condition, and the result found that knocking down METTL3 significantly inhibited the whole metastasis of PC-3 cells at the 4th week and 6th week (Fig. 5i). The metastatic organs were anatomized and imaged using the in vivo imaging system, which helped to locate and identify the metastases (Fig. 5j). The above metastatic tissues were made into slides and stained with H&E to locate the metastasis sites (Fig. 5k). In conclusion, knocking down METTL3 dramatically inhibited the tumor growth and metastasis in vivo.

### YTHDF2 mediates the mRNA degradation of LHPP and NKX3–1 in m^6^A-dependent way

**Fig. 6:**
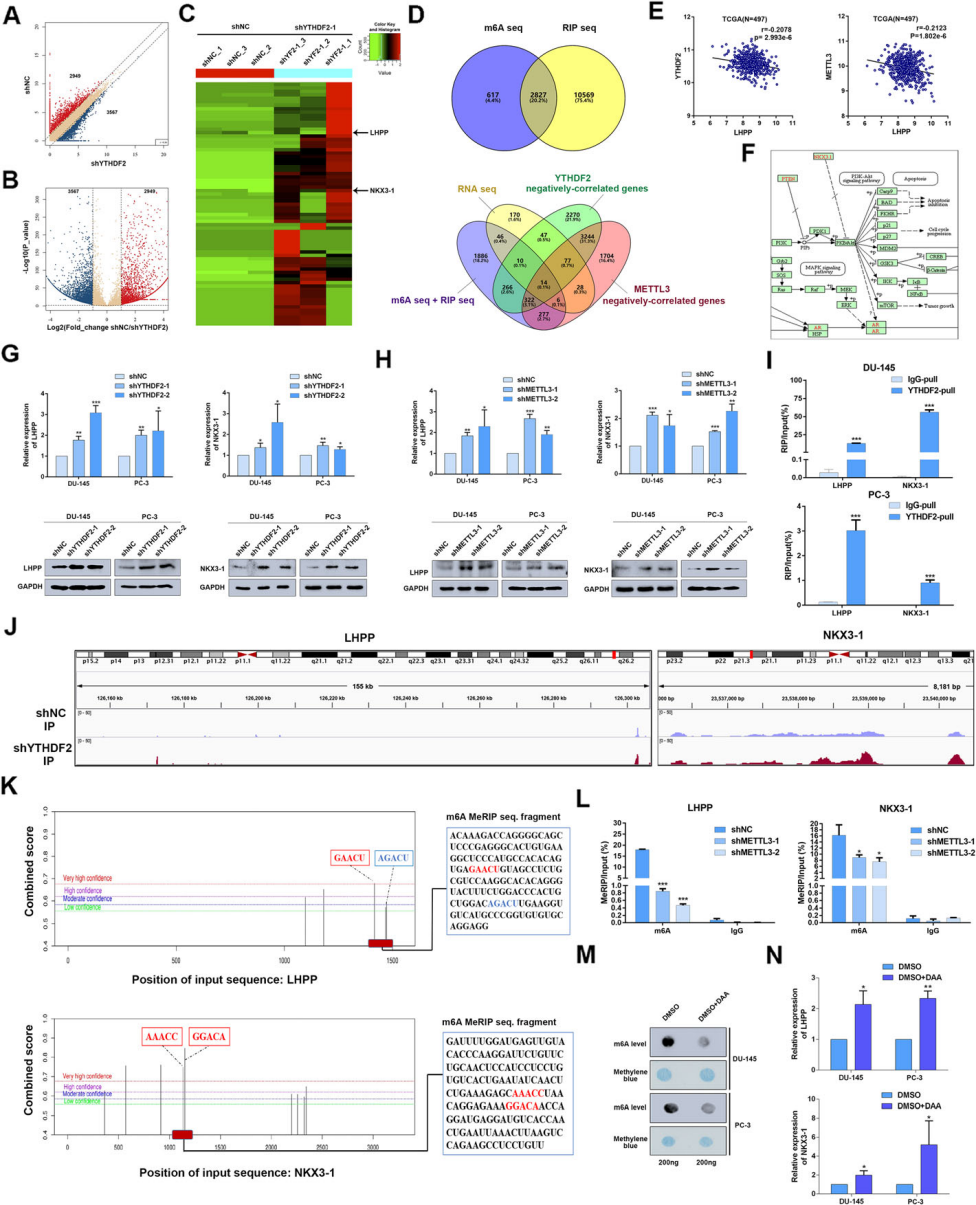
YTHDF2 mediates the mRNA degradation of LHPP and NKX3–1 in m^6^A-dependent way. **a**-**c** MeRIP-seq data in two groups (shNC and shYTHDF2, three triplicates respectively). **a** Scatter plot of differentially methylated genes. The values of X and Y axes in the scatter plot are the averaged RPM (reads per million) values of each group (log_2_ scaled). Genes above the top line (2949 red dots, upregulation in shNC group) or below the bottom line (3567 blue dots, upregulation in shYTHDF2 group) indicate more than 2-fold change between two compared groups. Brown dots indicate methylation level without differentially expression. **b** Volcano plot of differentially methylated genes. The values of X and Y axes in the volcano plot are the fold change (log_2_ transformed) and *P* value (−log_10_ transformed) between two groups, respectively. Red/Blue dots indicate 2-fold change differentially methylated genes with statistical significance (3567 blue dots, upregulated in shYTHDF2 group, 2949 red dots, upregulated in shNC group). Brown circles indicate non-differentially methylated gene. **c** Heatmap analyzed from MeRIP-seq data listed the representative upregulated genes in m^6^A levels after knocking down YTHDF2, black arrow referred to the candidate target genes discussed below. **d** Venn diagram was plotted to show the intersected genes from MeRIP-seq, RIP-seq, mRNA-seq and YTHDF2/METTL3 negatively-correlated genes. Fourteen common genes were screened out. **e** The correlation between LHPP and YTHDF2 or METTL3 was plotted with GraphPad prism 6.0 by analyzing the negatively-correlated genes downloaded from LinkedOmics (TCGA data). And *r* = − 0.2078 (*P* = 2.993e-6) or − 0.2123 (*P* = 1.802e-6) separately. **f** The prostate cancer pathway involved in KEGG analysis was obtained from DAVID tool by inputting the 3567 upregulated genes in shYTHDF2 group in MeRIP-seq results as gene list. The graph was downloaded from KEGG database. **g** and **h** The mRNA and protein levels of LHPP and NKX3–1were detected with RT-qPCR and western blot after knocking down YTHDF2 or METTL3. GAPDH was the internal reference. Data were analyzed with student’s t test. **i** RIP-RT-qPCR was utilized to confirm the LHPP and NKX3–1 mRNA enrichment by YTHDF2 in DU-145 and PC-3 cell lines. Data were analyzed with student’s t test. **j** The mapped reads represent enriched RNA fragments by MeRIP experiment. RNA methylation profiles were loaded in IGV and m^6^A modification peak alterations in LHPP and NKX3–1 mRNA full length were visualized. **k** The potential m^6^A sites of LHPP and NKX3–1 predicted by SRAMP were combined and co-localized with m^6^A MeRIP-seq results. Different color lines indicated different confidences (red, purple, blue and green respectively represents very high, high, moderate and low confidence). The sequence beside is the fragments captured in MeRIP-seq, which was co-localized with predicted sites. **l** MeRIP-RT-qPCR was used to detect the m^6^A level alterations of LHPP and NKX3–1 after knocking down METTL3 in PC-3 cell line. Data was analyzed with student’s t test. **m** The total RNA m^6^A level after treatment of DAA was detected with m^6^A RNA dot-blot assay and compared with DMSO treatment. Methylene blue staining was loading control. **n** RT-qPCR was utilized to detect the mRNA level of LHPP and NKX3–1 after DAA treatment. Data was analyzed with student’s t test. Error bars represent the SD obtained from at least three independent experiments; *P ≤ 0.05, **P ≤ 0.01, ***P ≤ 0.001

As previous work reported, YTHDF2 could induce the target mRNAs degradation by reading the m^6^A modification sites [7]. We assumed that YTHDF2 similarly degraded some target mRNAs to induce pAKT. To investigate the potential and direct m^6^A-modified targets of YTHDF2, we conducted a MeRIP-seq in YTHDF2 stably knocked down PC-3 cell line (PC-3 shYTHDF2) and its control (PC-3 shNC). The scatter plot and volcano plot indicated that the m^6^A levels of 3567 genes were upregulated and 2949 genes were downregulated after knocking down YTHDF2 (Fig. 6a, b). The representative genes upregulated in m^6^A levels were presented as a heatmap (Fig. 6c), which included the candidate targets (black arrow indicated) discussed as follows. Combining the RIP-seq data from previous study [7], we found the overlap with our MeRIP-seq, and finally identified 2827 common genes. In addition to MeRIP-seq, mRNA-seq after knocking down YTHDF2 in PC-3 cell line was also conducted. The LinkedOmics online database [25] was used to obtain the gene lists that were negatively associated with YTHDF2 and METTL3. By analyzing 2827 common genes, mRNA-seq, negatively-associated gene lists, we found 14 common genes at last (Fig. 6d). Through a literature review, we found that LHPP was reported as a tumor suppressor and negatively with pAKT (S473) in hepatocellular carcinoma (HCC) [26], and overexpression of LHPP downregulated pAKT to inhibit the proliferation and migration of cervical cancer [27], which is consistent with the phenotype induced by YTHDF2 and METTL3 in PCa. To investigate the regulatory relationship between YTHDF2 and LHPP, and the specific mechanisms of LHPP in PCa, we firstly detected their correlation in LinkedOmics online database, which suggested that LHPP was negatively correlated with both YTHDF2 (r = − 0.2078, *P* < 0.001) and METTL3 (r = − 0.2123, *P* < 0.001) (Fig. 6e). In addition, we analyzed all m^6^A-significantly-upregulated genes in YTHDF2-knock-down PC-3 cell lines with the DAVID tool [28, 29]. Interestingly, the achieved KEGG pathway results revealed that “pathway of prostate cancer” including well-known tumor suppressor NKX3–1 was also predicted to be involved in PCa (Fig. 6f). NKX3–1 was previously reported to be a crucial inhibitor of AKT phosphorylation. Thus, we tested whether YTHDF2 could downregulate both LHPP and NKX3–1 to indirectly inhibit pAKT (S473 and T308) by RT-qPCR and western blot assays. We found that both the mRNA level and protein level of LHPP and NKX3–1 were significantly upregulated after knocking down YTHDF2 in both DU-145 and PC-3 cell lines (Fig. 6g). Furthermore, rescue experiment also confirmed that knock-down of YTHDF2 partially rescued the reduced expression of NKX3–1 and LHPP by siNKX3–1 and siLHPP at protein levels (supplementary figure 8 A-C). Of note, we further observed that knocking down METTL3 consistently upregulated expression of LHPP and NKX3–1 at both mRNA and protein levels (Fig. 6h). RIP-RT-qPCR was also conducted, and the results validated that the YTHDF2 antibody obviously enriched the mRNA of LHPP and NKX3–1 compared to the IgG pull-down group in the DU-145 and PC-3 cell lines (Fig. 6i), which confirmed the direct interaction between YTHDF2 and two targets. In addition, the protein levels of LHPP and NKX3–1 were also confirmed to be upregulated in IHC staining of mice subcutaneous xenografts after knocking down YTDHF2 or METTL3 (supplementary figure 9A and B). The obvious m^6^A peak elevation of LHPP and NKX3–1 full length induced by knock-down of YTHDF2 was totally visualized with IGV (Fig. 6j). Furthermore, online m^6^A sites prediction tool SRAMP (http://www.cuilab.cn/sramp) [30] was also used, and separate two m^6^A sites in 3’UTR of LHPP and NKX3–1 at single site resolution were identified, which was well-matched and overlapped with significantly-upregulated m^6^A sites (fragments) in the YTHDF2 knock-down PC-3 cell line from MeRIP-seq data analysis (Fig. 6k). Further dual luciferase activity assay also indicted that knock-down of YTHDF2 significantly elevated the luciferase activity in wild type group (m^6^A sites included 3’UTR) but not in mutated group (mutated m^6^A sites from A to T), and vice versa (overexpression of YTHDF2) (supplementary figure 10A-C). To investigate whether this regulation was m^6^A-dependent, MeRIP-RT-qPCR was performed with the matched m^6^A sites (sequence). The results indicated that the m^6^A specific antibody enriched m^6^A-modified mRNA of LHPP and NKX3–1 mRNA compared to the IgG negative control, and furthermore, knocking down METTL3 significantly reduced the m^6^A-modified mRNA enrichment of LHPP and NKX3–1 mRNA than shNC group in PC-3 cell line (Fig. 6l). Catalytic-dead METTL3 was also constructed by mutating wildtype METTL3 (395 to 398, DPPW to APPA) (supplementary figure 11A) to observe the function and the expression of two targets after its transfection. The result revealed that the expression of LHPP and NKX3–1 was significantly inhibited by overexpression of wildtype METTL3 but not catalytic-dead METTL3 (supplementary figure 11B). Furthermore, overexpression of catalytic-dead METTL3 couldn’t upregulate the m6A level or promote PCa cell proliferation and migration like wildtype METTL3 (supplementary figure 11C, D and E). In addition, we also used the global methylation inhibitor, 3-deazaadenosine (DAA) to treat PCa cell lines and found that the total m^6^A levels detected with RNA m^6^A dot-blot assay were remarkably reduced compared to the DMSO control group at 200 ng RNA concentration (Fig. 6m). Consistently, the mRNA expression of LHPP and NKX3–1 was also upregulated with the same concentration DAA treatment (Fig. 6n). Taken together, YTHDF2 mediated the mRNA degradation of LHPP and NKX3–1 by reading m^6^A modified sites, and this regulation was m^6^A-dependent.

### The tumor suppressor role of LHPP and NKX3–1 in PCa

**Fig. 7:**
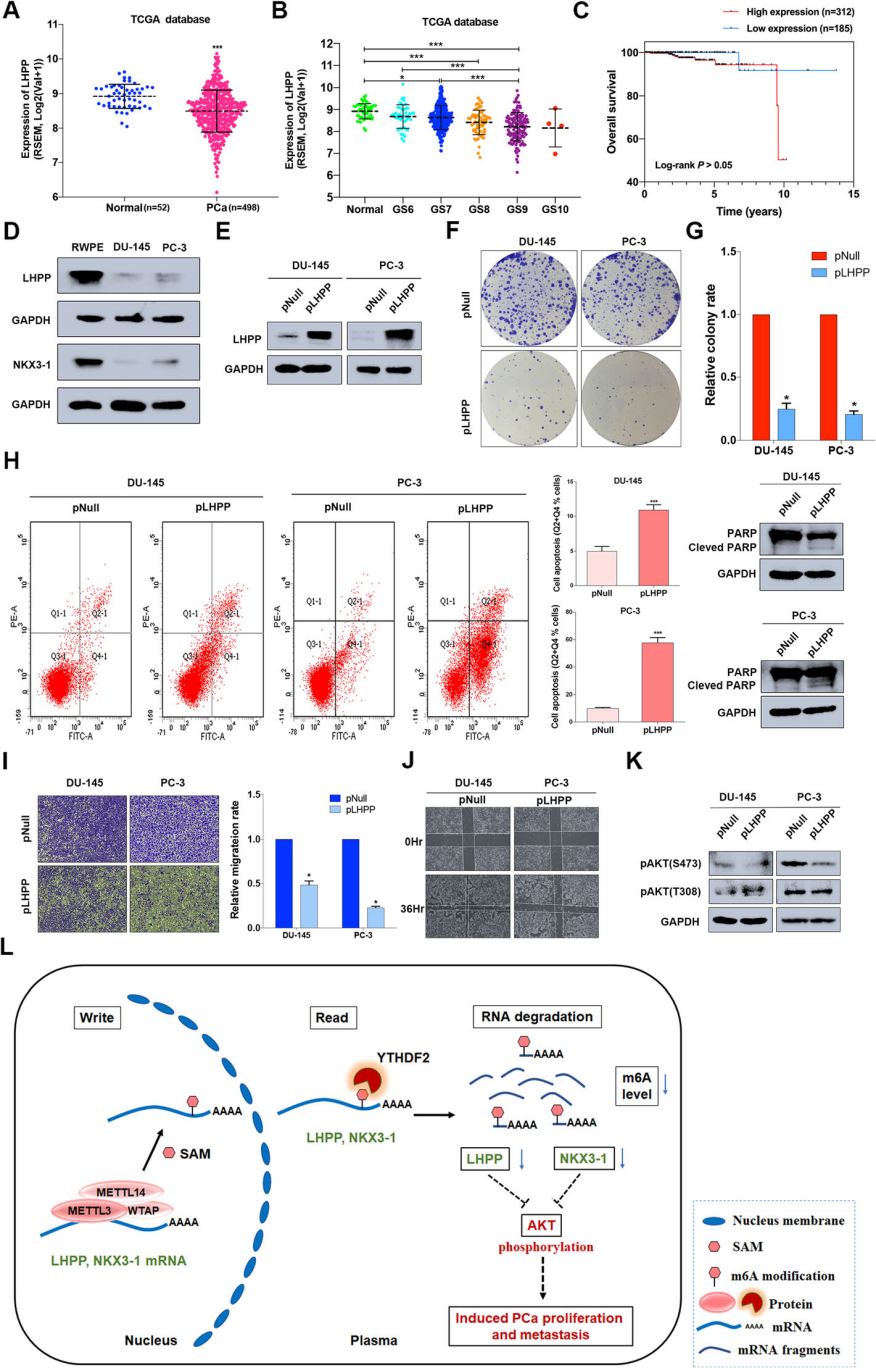
The tumor suppressor role of LHPP and NKX3–1 in PCa. **a**, **b** The expression pattern of LHPP in total or in separate Gleason score of 498 PCa tissues (52 normal controls) were plotted with TCGA database. Student’s t test was used for the statistical analysis of two groups comparison. One-way ANOVA test with Bonferroni’s correction was used for statistical analysis of more than two groups comparisons. **c** The survival probability of LHPP was determined with Kaplan–Meier analysis and log-rank test in 497 PCa patients according to the relative expression of LHPP. **d** The expression of LHPP and NKX3–1 in RWPE-1 and PCa cell lines were identified by western blot. GAPDH was the internal reference. **e** The overexpression efficiency of pLHPP plasmid (transient transfection with FuGENE HD Transfection Reagent, 0.5 μg/ml) was validated by western blot assay. GAPDH was the internal reference. **f** and **g** The colony formation assay (representative wells were presented) was used to evaluate the colony rate after overexpressing LHPP. Mann-Whitney test was used for the statistical analysis. **h** Flow cytometry assay (representative images were presented) and western blot assay were used to evaluate the apoptosis analysis induced by overexpressing LHPP. GAPDH was the internal reference. **i** and **j** Trans-well assay and wound-healing assay (representative wells were presented) were used to determine the cell migration after overexpressing LHPP. Mann-Whitney test was used for the statistical analysis. **k** The AKT phosphorylation inhibited by LHPP was analyzed by western blot assay. GAPDH was the internal reference. **l** All the findings in this study is concluded as a schematic diagram. Error bars represent the SD obtained from at least three independent experiments; *P ≤ 0.05, **P ≤ 0.01, ***P ≤ 0.001

NKX3–1 has been reported as a tumor suppressor, and loss of NKX3–1 contributed to prostate carcinogenesis and tumor progression [31–33]. The forced expression of NKX3–1 significantly inhibited PCa cell proliferation and migration [34–36]. Furthermore, NKX3–1 attenuated the ability of AKT by suppressing its phosphorylation [37, 38]. E-cadherin was also restored by NKX3–1 to abrogate EMT progression [36]. In this study, we found that knocking down both YTHDF2 and METTL3 induced NKX3–1 and LHPP expression and inhibited AKT phosphorylation, which was consistent with NKX3–1 functions in PCa. However, studies about LHPP are relatively rare especially in PCa. In this section, we focused on the role of LHPP in PCa. To begin with, TCGA database was used to detect the expression pattern of LHPP in PCa. The results indicated that LHPP was downregulated in PCa tissues (*n* = 498) compared with the normal controls (*n* = 52) (*P* < 0.001) and had a negative correlation with Gleason scores (Fig. 7a and b). Subgroup analysis also indicated that LHPP was significantly downregulated in higher stages and lymph node metastasis (pN1 vs. pN0) patients (supplementary figure 12A and B). However, we could not find a prognostic value of LHPP expression and overall survival (*P* > 0.05) (Fig. 7c). The protein level tested by western blot assay also revealed that LHPP (and also NKX3–1) was extremely low in PCa cell lines than RWPE-1 cell line (Fig. 7d). The above data indicated that LHPP was a potential tumor suppressor in PCa. To further identify the function of LHPP in PCa, we used the overexpression plasmid (pLHPP) to force the expression of LHPP in PCa cell lines (Fig. 7e). The colony formation assay suggested that overexpression of LHPP dramatically inhibited the colony formation rate (Fig. 7f and g). The apoptosis analysis with flow cytometry showed that overexpression of LHPP induced the apoptosis in DU-145 and PC-3 cell lines, which was confirmed by the elevated cleaved PARP1 by western blot (Fig. 7h). The trans-well assay and wound-healing assay were also performed to evaluate the effect of LHPP on the migration abilities of DU-145 and PC-3 cell lines. The results suggested that the forced expression of LHPP obviously inhibited the migration abilities of PCa cell lines (Fig. 7i and j). Rescue experiments (between YTHDF2 and LHPP/NKX3–1) also revealed the consistent phenotypes (supplementary figure 8 D-G). In addition, we also established a xenograft mouse model to further confirm the in vitro rescue experiment result. And the results indicated that knock-down of NKX3–1 and LHPP could significantly induced the tumor growth, but knock down of YTHDF2 or METTL3 partially rescued the mice tumor growth induced by knock down of LHPP and NKX3–1 (supplementary figure 13 A, B and C). Interestingly, the western blot assay also found that AKT phosphorylation (S473) could be inhibited by overexpressed LHPP (Fig. 7k), which was also consistent with knock-down of YTHDF2 or METTL3, and supported by previous studies [26, 27]. KEGG analysis of LHPP and NKX3–1 common negatively correlated genes (supplementary figure 14A) also indicated that the most significant pathways including PI3K-AKT signaling pathway and several crucial pathways like ‘pathways in cancer’ and etc. were involved (supplementary figure 14B). To further validate the indirect AKT phosphorylation regulation of YTHDF2 via LHPP and NKX3–1, AKT inhibitor (MK-2206, Selleck) was used to treat PCa cell lines combined with pYTHDF2 plasmid transfection. The result indicated that overexpression of YTHDF2 rescued the inhibited pAKT (S473 and T308) level by MK2206 (Supplementary figure 15). In conclusion, both LHPP and NKX3–1 are tumor suppressors to inhibit AKT phosphorylation in PCa progressions. Thus, in this study we found that YTHDF2 as a crucial m^6^A reader exerted its degradation function by targeting two tumor suppressors LHPP and NKX3–1, consequently inducing AKT phosphorylation in PCa. All of these results are concluded as a schematic diagram (Fig. 7l).

## Discussion

Although emerging evidence of m^6^A has been reported in different fields, the specific mechanisms of m^6^A involved in different tumor progressions still remain elusive. Writers produce m^6^A modifications while readers mediate different biological processes and events by binding to m^6^A modified sites. YTHDF2 belonging to one crucial reader was the first to be validated to degrade target mRNAs or non-coding RNAs by binding to m^6^A modified sites with the conserved core motif of G(m^6^A) C to realize target-deficient phenotypes [7]. Specifically, the C-terminal domain of YTHDF2 selectively binds to m^6^A-modification sites whereas the N-terminal domain mediates the localization of the YTHDF2-mRNA complex to cellular RNA decay sites by recruiting the CCR4-NOT complex [7, 17]. Researchers have utilized three Cre/LoxP systems in vivo to validate that YTHDF2 in hematopoietic stem cells facilitated the decay of m^6^A-modified mRNAs of Wnt target genes, contributing to the repression of Wnt signaling at a steady state [19]. Another finding indicated that YTHDF2 modulated the neural development in mice by mediating the clearance of a series of m^6^A-modified neuron differentiation-related mRNAs [39]. In addition, YTHDF2 were also found to delay the entry of cells into G2 cell cycle by degrading m^6^A-containing mRNAs of CDK2 and CCNA2, consequently suppressing adipogenesis [40]. Recently the regulatory role of YTHDF2 involved in human cancers also loomed up. It has been confirmed that YTHDF2 induced the targeted degradation of the tumor suppressor SOCS2 to promote tumor progressions of HCC, and this regulation was dependent on METTL3-induced m^6^A modifications [20]. Until very recently, more and more researches reported that YTHDF2 induced decay of targeted mRNAs was involved in several tumor progressions. YTHDF2 reduction fuels inflammation and vascular abnormalization by decaying m^6^A-containing IL11 and SERPINE2 mRNAs in HCC [41]. YTHDF2 was frequently overexpressed in AML, and the m^6^A modified apoptosis-induced gene TNFRSF2 was targeted by YTHDF2 to decrease its half-life, which was identified as a unique therapeutic target [42]. However, the function and specific mechanisms of YTHDF2 in PCa are still unelucidated.

In this study, we focused on the role of YTHDF2 and its upstream cooperator METTL3 in PCa. YTHDF2 and METTL3 were confirmed to be frequently upregulated in PCa, and their high expression predicted a poor survival. Both in vivo and in vitro experiments indicated that knocking down YTHDF2 or METTL3 markedly inhibited the PCa cell proliferation and migration accompanying with inhibited AKT phosphorylation. To investigate the potential degradation targets of YTHDF2, MeRIP-seq, mRNA-seq and database analysis were all applied, and several candidate genes were identified. Through further experiments confirmation, LHPP and NKX3–1 were identified as the primary targets of YTHDF2. YTHDF2 could directly bind to m^6^A containing of LHPP and NKX3–1 to induce mRNA decay. To confirm this regulation is m^6^A-dependent, we also investigated the molecular mechanism of METTL3 in regulating YTHDF2-targeted LHPP and NKX3–1 mRNA. Consistently, knocking down METTL3 significantly enriched less m^6^A containing mRNA fragments of LHPP and NKX3–1 than shNC control accompanying with the elevated expression level of LHPP and NKX3–1. Additionally, demethylation treatment with DAA to PCa cell lines also induced the similar expression of LHPP and NKX3–1 with METTL3-dificiency, which further demonstrated above regulation was m^6^A-dependent. Thus, we speculated that YTHDF2 induced the degradation of LHPP and NKX3–1 by binding to the m^6^A modified sites mediated by METTL3.

To illustrate the mechanism of YTHDF2 and METTL3 induced pAKT signaling, we demonstrated it through the mechanism of their common targets LHPP and NKX3–1. LHPP is a histidine phosphatase and reported to function as the tumor suppressor role in HCC and cervical cancer, and it modulated tumor progression by inhibiting AKT phosphorylation [26, 27]. In this study, we first detected the low expression of LHPP in PCa patients and cell lines. The forced expression of LHPP markedly suppressed the cell proliferation and migration. Interestingly, AKT phosphorylation (S473) was also inhibited by the overexpression of LHPP which was consistent with other studies described as above. Another target NKX3–1 was a publicly-recognized crucial tumor suppressor in PCa. It is convincing that overexpression of NKX3–1 inhibited the proliferation and migration [34, 35]. In addition, AKT phosphorylation (T308) could also be abrogated by NKX3–1 in AR-deleted PCa cell lines (like PC-3) [37, 43]. Herein, we investigated the role of post-transcriptional modification (m^6^A) in regulating its expression. Considering that both LHPP and NKX3–1 can inhibit the AKT phosphorylation to restrain PCa progressions, we may suspect that YTHDF2 promotes the tumor progression via degrading LHPP and NKX3–1 to upregulate pAKT (S473 and T308).

## Conclusions

In summary, we provided in vitro and in vivo evidence to elucidate that YTHDF2 mediates the mRNA degradation of the tumor suppressors LHPP and NKX3–1 in m^6^A-depnedent way to regulate AKT phosphorylation-induced tumor progression in prostate cancer. We hope this study will provide a novel regulatory mechanism that may assist the development of potential diagnosis biomarkers or therapeutic targets of PCa in the future.

## Supplementary information

**Additional file 1.**

**Additional file 2 **: **Table S**1**.** The core interference sequences of shRNAs. **Table S2.** The primers used in this study.

**Additional file 3: Figure S1.** Cohorts from Oncomine database indicate both YTHDF2 and METTL3 are significantly upregulated in PCa. **(**A) Expression pattern of YTHDF2 in Grasso Prostate cohort. (B)-(F) Expression pattern of METTL3 in Arredouani Prostate, Luo Prostate, Wallace Prostate, Singh Prostate, Welsh Prostate cohorts. Student’s t test was used for statistical analysis.

**Additional file 4 **: **Figure S2.** Expression pattern of m^6^A associated genes in PCa cell lines. (A) YTHDF2 mRNA expression (RNAseq data) in several PCa cell lines from CCLE database (B) Western blot assay. The protein levels of METTL14, WTAP, FTO and ALKBH5 in PCa cell lines compared with normal prostate cell line (RWPE). GAPDH was the internal reference. (C)-(F) mRNA levels of METTL14, WTAP, FTO and ALKBH5 (RNAseq data) in several PCa cell lines from CCLE database.

**Additional file 5: Figure S**3**.** Expression pattern and Kaplan–Meier analysis of m^6^A associated genes in TCGA database. (A)-(D) Expression pattern of METTL14, WTAP, FTO and ALKBH5 in PCa tissues of TCGA database. METTL14, FTO and ALKBH5 were all downregulated in PCa tissues compared with normal tissues. However, no difference of WTAP expression was observed between PCa and normal tissues. Student’s t-test was used for statistics analysis. (E)-(H) The expression levels of above genes were not significantly correlated with PCa overall survival rate. Kaplan–Meier curve (log-rank test) was used for statistics analysis. *P ≤ 0.05, **P ≤ 0.01, ***P ≤ 0.001.

**Additional file 6: Figure S**4**.** YTHDF2 promotes EMT progression via pAKT/GSK3β/SNAIL pathway. Western blot analysis. Forced expression of YTHDF2 upregulated the pGSK3β and SNAIL expression in both DU-145 and PC-3 cell lines.

**Additional file 7: Figure S5.** Overexpression of wild type YTDHF2 or METTL3 rescues the expression and cellular biofunction. (A) Western blot assay. The protein levels of YTHDF2 and METTL3 were rescued by wild type overexpression plasmid in DU-145 cell line. (B)-(C) Colony formation and trans-well assy. Cell colony ability and migration ability were rescued by wild type overexpression of YTHDF2 or METTL3 after knock-down in DU-145 cell line.

**Additional file 8: Figure S6.** 5’UTR targeting siRNA of METTL3 and 3’UTR targeting siRNA of YTHDF2 consistently knock down endogenous expression and inhibited the cell migration. (A) Western blot assay. The knock-down effect of UTR targeting siRNAs. (B) Trans-well assay. Cell migration ability was inhibited after knock-down of YTHDF2 and METTL3 with UTR targeting siRNAs.

**Additional file 9: Figure S7.** Knocking down METTL3 has no effect on the expression of FTO and ALKBH5. (A) Western blot assay. Knocking down METTL3 didn’t change the expression of FTO or ALKBH5 at protein levels. (B) RNA m^6^A Dot-blot assay. Overexpression of FTO or ALKBH5 induced a slight downregulation of m6A levels in PCa cell lines at 200 ng total RNA concentration. (C) Western blot assay. The overexpression efficiencies of FTO and ALKBH5 were identified in PCa cell lines.

**Additional file 10: Figure S8.** Rescue experiments between YTHDF2 and LHPP/NKX3–1/pAKT. (A)-(C) The protein level changes of rescue experiments in PC-3 cell line. (A) Western blot assay. The suppressed expression of NKX3–1 induced by siNKX3–1-pool was partially restored by knock-down of YTHDF2 (siYTHDF2-pool) at protein level. (B) Western blot assay. The suppressed expression of LHPP induced by siLHPP-pool was partially restored by knock-down of YTHDF2 (siYTHDF2-pool) at protein level (C) Western blot assay. Knock-down of LHPP partially rescued the expression of pAKT(s473) at protein level reduced by knock-down of YTHDF2. (D)-(G) The phenotypes induced by rescue experiments. (D) Colony formation assay (representative wells were presented). Colony formation rate promoted by siNKX3–1-pool was partially inhibited by knocking down YTHDF2. (E) Trans-well assay (representative wells were presented). The migration ability promoted by siNKX3–1-pool was partially inhibited by knocking down YTHDF2. The photograph was taken under 20× objective (10 × 20). (F) Colony formation assay (representative wells were presented). Colony formation rate promoted by siLHPP-pool was partially inhibited by knocking down YTHDF2. (G) Trans-well assay (representative wells were presented). The migration ability promoted by siLHPP-pool was partially inhibited by knocking down YTHDF2. The photograph was taken under 20× objective (10 × 20).

**Additional file 11: Figure S9.** IHC staining of LHPP and NKX3–1 in subcutaneous xenografts after knocking down of YTHDF2 or METTL3. (A)-(B) Representative IHC staining micrographs of LHPP, NKX3–1 in tumor xenografts were conducted. Scale bar = 100 μm.

**Additional file 12: Figure S10.** Mutated m^6^A sites inhibited the binding of YTHDF2 to LHPP and NKX3–1. (A) The schematic diagram presented the dual luciferase vector pmirGlo and inserted wildtype and mutated sequences (A to T). (B) Dual luciferase activity assay. Overexpression of YTHDF2 inhibited the luciferase activity of wild type LHPP or NKX3–1 but not of the mutated in PC-3 cell line. Student’s t-test was used for statistics analysis. (C) Knock-down of YTHDF2 (siYTHDF2-pool) consistently elevated the luciferase activity of wild type LHPP or NKX3–1 but not of the mutated in PC-3 cell line. Error bars represent the SD obtained from at least three independent experiments and student’s t-test was used for statistics analysis. *P ≤ 0.05, **P ≤ 0.01, ***P ≤ 0.001.

**Additional file 13: Figure S11.** Catalytic dead METTL3 loses the function of m6A formation, targets inhibition and tumor progression promotion. (A) Schematic diagram. The catalytic region (359 to 398, DPPW) of wild type METTL3 was mutated into APPA. (B) Western blot assay. The expression of LHPP and NKX3–1 were downregulated after wild type transfection but not mutated type. (C) RNA m6A dot-blot assay. Overexpression of catalytic dead METTL3 couldn’t promote total m^6^A level like wild type METTL3. (D) and (E) Catalytic dead METTL3 has little effect on proliferation and migration.

**Additional file 14: Figure S12.** Subgroup expression pattern of LHPP in TCGA database. (A) Clinical stage analysis. LHPP had lower expression in upregulated stages. One-way ANOVA with multiple comparison test (Bonferoni correction) was used for statistics analysis. (B) Lymph node metastasis analysis. LHPP was downregulated in PCa tissues with lymph node metastasis (pN1) compared with non-lymph nodes metastasis (pN0). Student’s t-test was used for statistics analysis. *P ≤ 0.05, **P ≤ 0.01, ***P ≤ 0.001.

**Additional file 15: Figure S13.** Knock down of YTHDF2 or METTL3 partially rescued the mice tumor growth induced by knock down of LHPP and NKX3–1. (A) The subcutaneous tumor models were observed in six different groups (blank arrows indicated tumor xenografts). (B) The BALB/c nude mice were sacrificed for the xenografts, and the size was measured by the beside ruler. (C) The tumor weight of each xenograft was measured and plotted.

**Additional file 16: Figure S14.** Multiple pathways associated with tumors are mainly involved in YTHDF2 mediated PCa progression. (A) The Venn diagram was used to present the common negatively-correlated genes (934) with LHPP and NKX3–1, which were downloaded from TCGA database and analyzed by LinkedOmics. (B) KEGG pathway analysis with the 934 common genes shown in (A) indicated that multiple pathways including ‘PI3K-AKT pathway’ and ‘JAK-STAT pathway’ etc. (blue arrow) were mainly involved in PCa tumor progression.

**Additional file 17: Figure S15.** Overexpression of YTHDF2 could significantly rescued the reduced AKT phosphorylation level by AKT inhibitor. Western blot assay. Forced expression of YTHDF2 significantly rescued the inhibited pAKT(S473) and pAKT(T308) level by AKT inhibitor (MK-2206) in both DU-145 and PC-3 cell lines.

## Data Availability

The MeRIP-seq data have been deposited in the Gene Expression Omnibus (GEO) database and the accession number is GSE129408. In addition, we also used other previous published GEO data to conduct further analysis like the RNA immunoprecipitation sequencing (RIP-seq) data from GSE49339 (combined analysis with our MeRIP-seq).

## Abbreviations

PCa: Prostate cancer
m^6^A: N6-methyladenosine
MeRIP: m^6^A RNA immunoprecipitation
CRPC: Castration-resistant PCa
IHC: Immunohistochemistry
IGV: Integrative Genomics Viewer
shRNAs: Small hairpin RNAs
CCLE: Cancer Cell Line Encyclopedia
