# Supplementary material for: YTHDF2 mediates the mRNA degradation of the tumor suppressors to induce AKT phosphorylation in N6-methyladenosine-dependent way in prostate cancer
Source: Mol Cancer. 2020 Oct 29;19:152. doi: 10.1186/s12943-020-01267-6 (PMC7599101; doi:10.1186/s12943-020-01267-6)
Supplement: Supplementary file 1 — Additional file 1. [file 12943_2020_1267_MOESM1_ESM.docx]

**Supplementary materials and methods**

**Reagents and transfection.** Small interference RNA (siRNA) pool (Genepharma, Shanghai, China) was used to knock down YTHDF2, LHPP and NKX3-1 separately (siYTHDF2-pool, siLHPP-pool and siNKX3-1-pool) via lipofectamine 2000 transfection. UTR targeting siRNAs were commercially obtained from (RiboBio company, Guangzhou) to knock down YTHDF2 and METTL3. The sequences are listed in supplementary table. Overexpression plasmid pALKBH5 was obtained from Genechem (Shanghai, China), and pFTO was obtained from GeneCopoeia (Guangzhou, China). FuGENE HD Transfection Reagent (Promega, Madison, USA) was used to transfect the overexpression plasmids according to the manufacturer’s protocol.

***In vitro* AKT inhibition treatment**

AKT inhibitor MK-2206 2HCl was commercially obtained from Selleck (Houston, USA), DU-145 and PC-3 cells were incubated with 5μM final concentration AKT inhibitor for 48 hours combined with 500ng pYTHDF2 plasmid transfection per well. Proteins were extracted for further western blot assay.

**Dual-luciferase reporter assay**

The wildtype and mutated sequence were chemically synthesized by Sangon (Shanghai, China) and then inserted into pmirGlo luciferase expression vector (Promega, Madison, USA) between the SacI and SalI sites. Student’s t-test was used for statistics analysis. We used T to replace the wildtype A in m^6^A sites to construct mutated type plasmids. All inserted sequences are listed in supplementary table 1, which were verified by sequencing. PC-3 cells seeded in 96-wells plate were co-transfected with 50ng constructed plasmids and 50nM si-YTHDF2-pool or 50ng pYTHDF2 plasmid (NC and pNull as control transfection) per well. Cells were harvested 48 h after the transfection. The relative luciferase activity was determined by the Dual-Glo luciferase assay kit (Promega, Madison, USA).

**Catalytic-dead METTL3 construction**

Catalytic-dead METTL3 plasmid was constructed by mutating the wild type METTL3 (395 to 398, DPPW to APPA). 500 cells per well for colony formation and 3×10^4^ cells for trans-well were seeded.

**Mouse xenograft rescue experiment**

2 × 10^6^ PCa cells suspended in 100 μl PBS were injected in the flank of male BALB/c nude mice (4 weeks old). The siRNA-pools were dissolved and diluted with 30-50ul lipofectamine per mouse and injected into the xenograft twice a week. All animal studies and manipulations were performed in compliance with the institutional guidelines approved by the First Affiliated Hospital, School of Medicine, Zhejiang University.
