# Supplementary material for: YTHDF2 mediates the mRNA degradation of the tumor suppressors to induce AKT phosphorylation in N6-methyladenosine-dependent way in prostate cancer
Source: Mol Cancer. 2020 Oct 29;19:152. doi: 10.1186/s12943-020-01267-6 (PMC7599101; doi:10.1186/s12943-020-01267-6)
Supplement: Supplementary file 2 — Additional file 2 : Table S1. The core interference sequences of shRNAs. Table S2. The primers used in this study. [file 12943_2020_1267_MOESM2_ESM.docx]

**Supplementary table 1. The core interference sequences of shRNAs.**

| Name | Sense (5’-3’) |
| --- | --- |
| shNC^a^ | TTCTCCGAACGTGTCACGT |
| shYTHDF2-1^a^ | CCTACTTACCCAGTTACTACA |
| shYTHDF2-2^a^ | GCTCTGGATATAGTAGCAATT |
| shMETTL3-1^a^ | CCUGCAAGUAUGUUCACUA |
| shMETTL3-2^a^ | GCUACCUGGACGUCAGUAU |
| siYTHDF2-pool-1 | GCCCAAUAAUGCAUAUACUTT |
| siYTHDF2-pool-2 | GCUCUGGAUAUAGUAGCAATT |
| siYTHDF2-pool-3 | GCGGGUCCAUUACUAGUAATT |
| siLHPP-pool-1 | CCCGGCUGAAGGUGAGGUUTT |
| siLHPP-pool-2 | GAGCAAGGCCUGCGACCAUTT |
| siLHPP-pool-3 | CCCAAACUGUGUGGUAAUUTT |
| siNKX3-1-pool-1 | CUUAUCUGUUGGACUCUGATT |
| siNKX3-1-pool-2 | CGCUAUAAGACUAAGCGAATT |
| siNKX3-1-pool-3 | CAGCUAUCCUUACUACCCATT |
| siYTHDF2-1(3’-UTR) | TGTCGTGAATGTTTCTTCA |
| siYTHDF2-2(3’-UTR) | GGCTTTGGTTGTGATTTCA |
| siYTHDF2-3(3’-UTR) | CTGACCTTTTGTTACTCAA |
| siMETTL3-1(5’-UTR) | CCTTATTCGAGAGGTGTCA |

a: lentivirus vector: hU6-MCS-Ubiquitin-Luc_firefly-IRES-puromycin

**Supplementary table 2. The primers used in this study.**

| Name^a^ | Sequence (5’-3’) |
| --- | --- |
| GAPDH-F | AAGGTGAAGGTCGGAGTCA |
| GAPDH-R | GGAAGATGGTGATGGGATTT |
| NKX3-1-F | CTGAGGCCTGGGAGTCTCTT |
| NKX3-1-R | AGCCCAAACCACAGAAAATG |
| LHPP-F | GAGGCTGGGATTTGACATCTC |
| LHPP-R | GAGCAGGTATGGTCGCAGG |
| ^b^Me-NKX3-1-F | ACTCCATCCTCCTGTGTCACTG |
| ^b^Me-NKX3-1-R | TGGTGACATCCTCATCCTGGT |
| ^b^Me-LHPP-F | GGCTCCCATGCCACACAGTG |
| ^b^Me-LHPP-R | CGGGCATGACACCTTCAAGTCTG |
| ^c^LHPP-Me-Wt-F | CCACTGTGAAGGCTCCCATGCCACACAGTGAGAACTGTAGCCTCTGCGTCCAAGGCACACAGGGTACTTTCTGGACCCACTGCTGGACAGACTTGAAGGTGTCATGCCCGGTGTGTGCAGGG |
| ^c^LHPP-Me-Wt-R | TCGACCCTGCACACACCGGGCATGACACCTTCAAGTCTGTCCAGCAGTGGGTCCAGAAAGTACCCTGTGTGCCTTGGACGCAGAGGCTACAGTTCTCACTGTGTGGCATGGGAGCCTTCACAGTGGAGCT |
| ^c^LHPP-Me-Mut-F | CCACTGTGAAGGCTCCCATGCCACACAGTGAGATCTGTAGCCTCTGCGTCCAAGGCACACAGGGTACTTTCTGGACCCACTGCTGGACAGACTTGAAGGTGTCATGCCCGGTGTGTGCAGGG |
| ^c^LHPP-Me-Mut-R | TCGACCCTGCACACACCGGGCATGACACCTTCAAGTCTGTCCAGCAGTGGGTCCAGAAAGTACCCTGTGTGCCTTGGACGCAGAGGCTACAGATCTCACTGTGTGGCATGGGAGCCTTCACAGTGGAGCT |
| ^c^NKX3-1-Me-Wt-F | CGTTCTGCAACTCCATCCTCCTGTGTCACTGAATATCAACTCTGAAAGAGCAAACCTAACAGGAGAAAGGACAACCAGGATGAGGATGTCACCAACTGAATTAAACTTAAGTCCAGAAGCCG |
| ^c^NKX3-1-Me-Wt-R | TCGACGGCTTCTGGACTTAAGTTTAATTCAGTTGGTGACATCCTCATCCTGGTTGTCCTTTCTCCTGTTAGGTTTGCTCTTTCAGAGTTGATATTCAGTGACACAGGAGGATGGAGTTGCAGAACGAGCT |
| ^c^NKX3-1-Me-Mut-F | CGTTCTGCAACTCCATCCTCCTGTGTCACTGAATATCAACTCTGAAAGAGCAATCCTAACAGGAGAAAGGACAACCAGGATGAGGATGTCACCAACTGAATTAAACTTAAGTCCAGAAGCCG |
| ^c^NKX3-1-Me-Mut-R | TCGACGGCTTCTGGACTTAAGTTTAATTCAGTTGGTGACATCCTCATCCTGGTTGTCCTTTCTCCTGTTAGGATTGCTCTTTCAGAGTTGATATTCAGTGACACAGGAGGATGGAGTTGCAGAACGAGCT |

a: F, forward primer; R, reverse primer.

b: Primers used in MeRIP qRT-PCR.

c: Sequences inserted in pmirGlo vector between SacI and SalI sites for the dual luciferase activity assay.
