# Supplementary figures and images for: YTHDF2 mediates the mRNA degradation of the tumor suppressors to induce AKT phosphorylation in N6-methyladenosine-dependent way in prostate cancer
Source: Mol Cancer. 2020 Oct 29;19:152. doi: 10.1186/s12943-020-01267-6 (PMC7599101; doi:10.1186/s12943-020-01267-6)

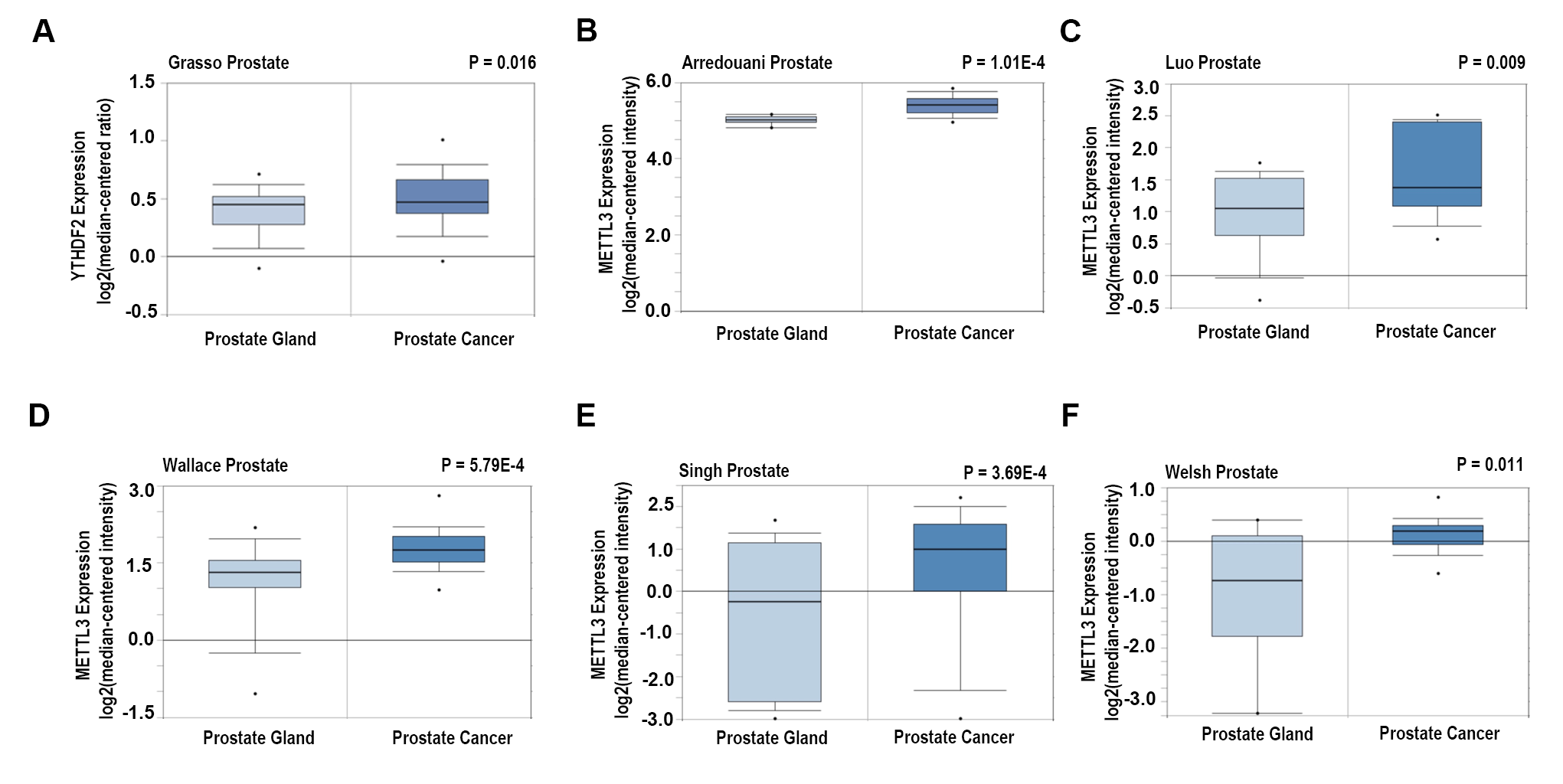

Supplement: Supplementary file 3 — Additional file 3: Figure S1. Cohorts from Oncomine database indicate both YTHDF2 and METTL3 are significantly upregulated in PCa. (A) Expression pattern of YTHDF2 in Grasso Prostate cohort. (B)-(F) Expression pattern of METTL3 in Arredouani Prostate, Luo Prostate, Wallace Prostate, Singh Prostate, Welsh Prostate cohorts. Student’s t test was used for statistical analysis. [file 12943_2020_1267_MOESM3_ESM.tif]

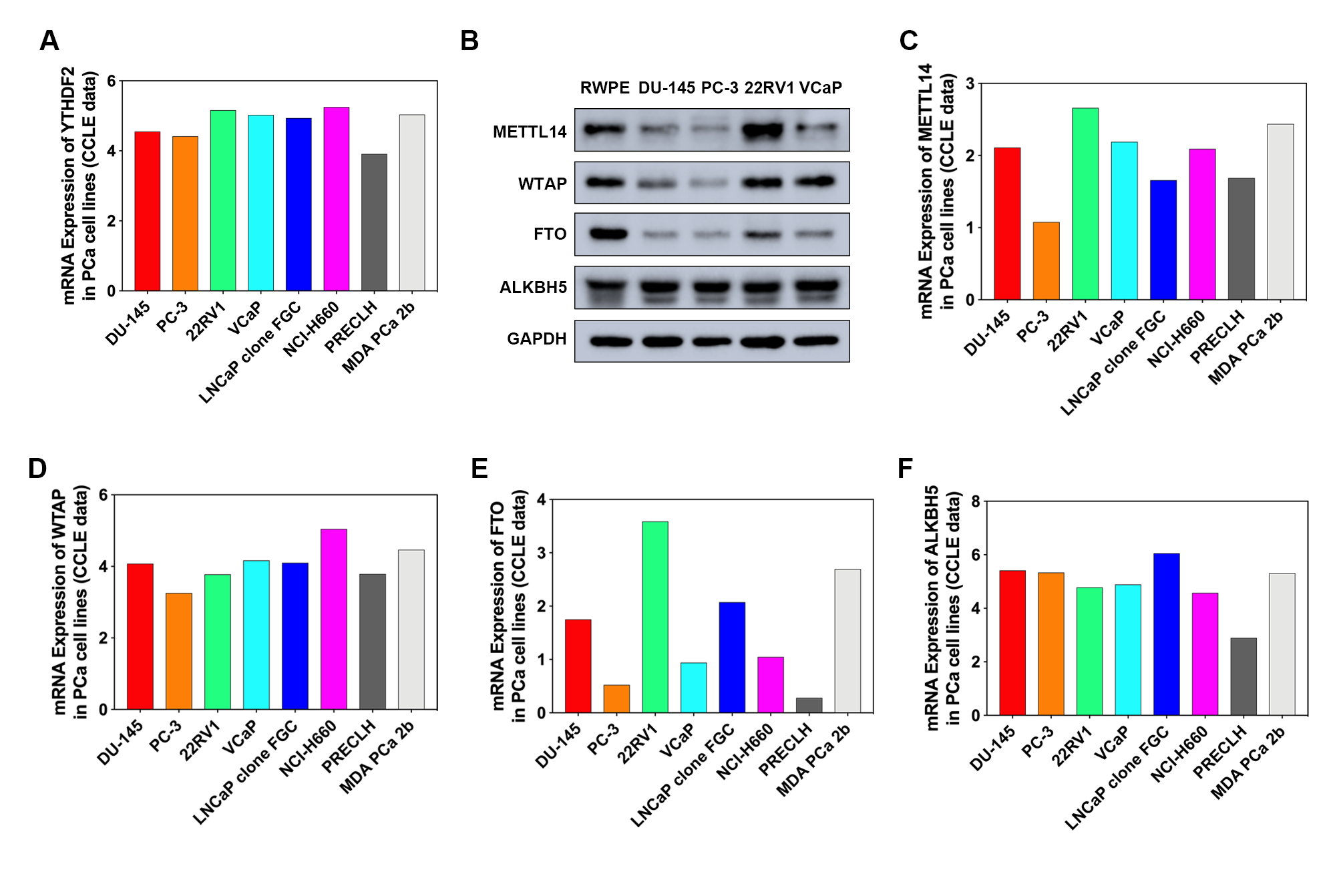

Supplement: Supplementary file 4 — Additional file 4 : Figure S2. Expression pattern of m6A associated genes in PCa cell lines. (A) YTHDF2 mRNA expression (RNAseq data) in several PCa cell lines from CCLE database (B) Western blot assay. The protein levels of METTL14, WTAP, FTO and ALKBH5 in PCa cell lines compared with normal prostate cell line (RWPE). GAPDH was the internal reference. (C)-(F) mRNA levels of METTL14, WTAP, FTO and ALKBH5 (RNAseq data) in several PCa cell lines from CCLE database. [file 12943_2020_1267_MOESM4_ESM.tif]

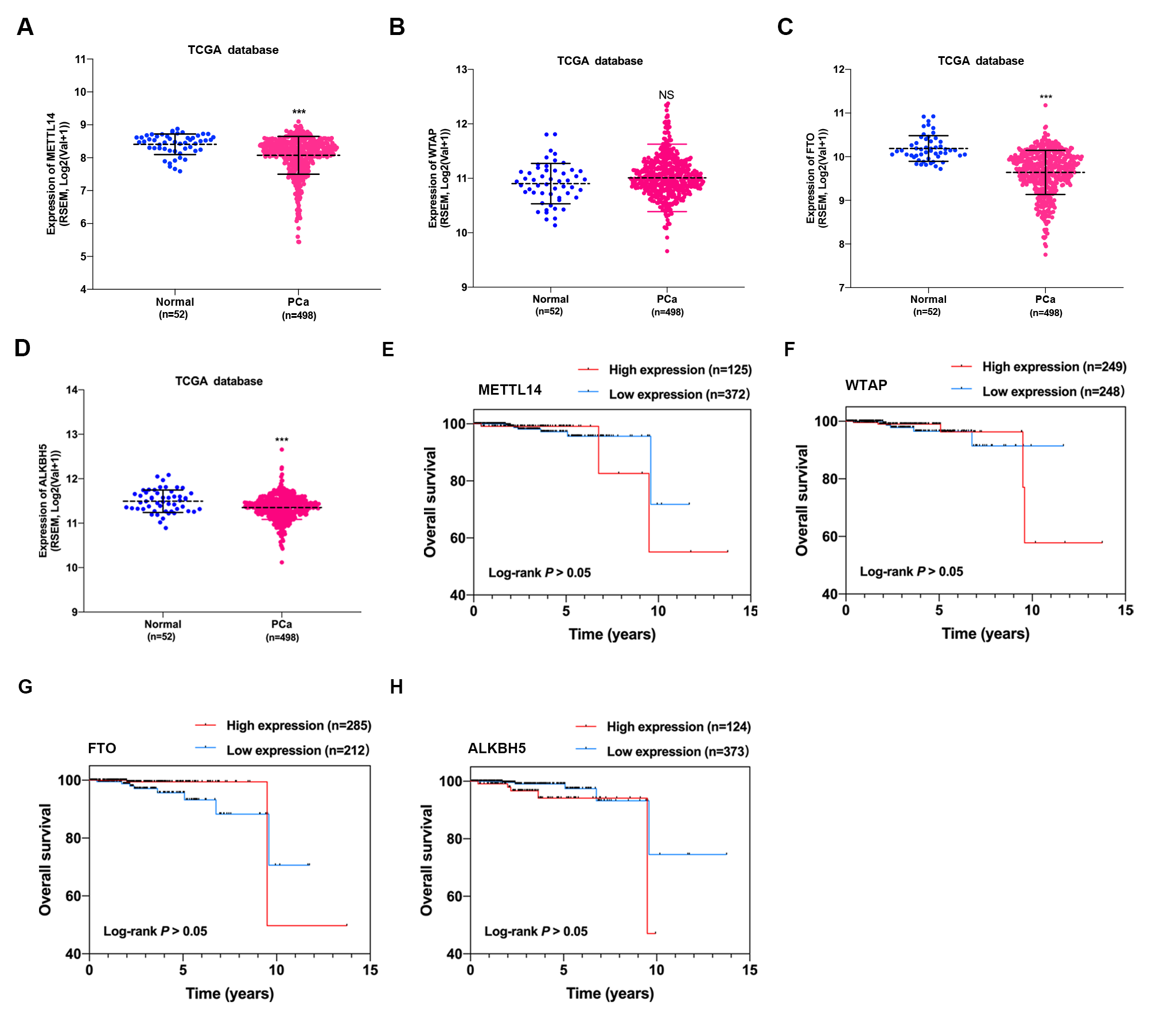

Supplement: Supplementary file 5 — Additional file 5: Figure S3. Expression pattern and Kaplan–Meier analysis of m6A associated genes in TCGA database. (A)-(D) Expression pattern of METTL14, WTAP, FTO and ALKBH5 in PCa tissues of TCGA database. METTL14, FTO and ALKBH5 were all downregulated in PCa tissues compared with normal tissues. However, no difference of WTAP expression was observed between PCa and normal tissues. Student’s t-test was used for statistics analysis. (E)-(H) The expression levels of above genes were not significantly correlated with PCa overall survival rate. Kaplan–Meier curve (log-rank test) was used for statistics analysis. *P ≤ 0.05, **P ≤ 0.01, ***P ≤ 0.001. [file 12943_2020_1267_MOESM5_ESM.tif]

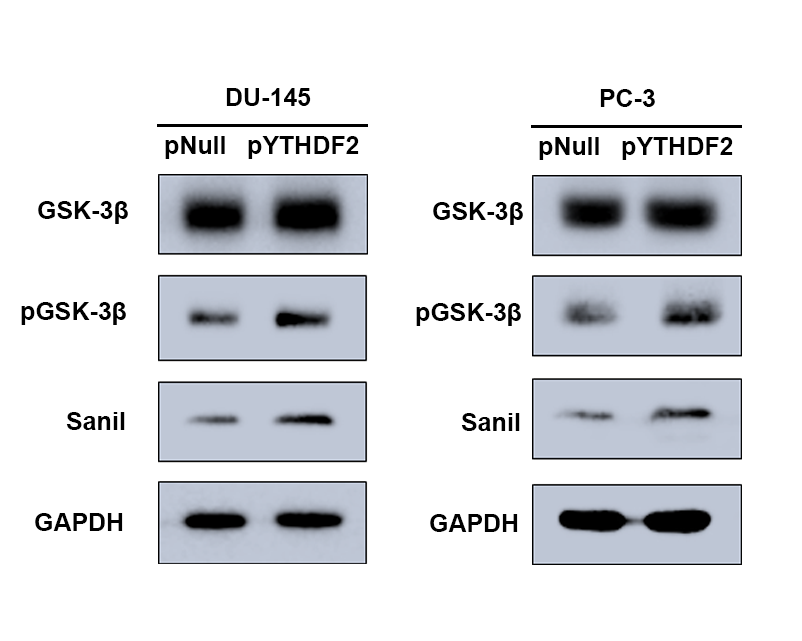

Supplement: Supplementary file 6 — Additional file 6: Figure S4. YTHDF2 promotes EMT progression via pAKT/GSK3β/SNAIL pathway. Western blot analysis. Forced expression of YTHDF2 upregulated the pGSK3β and SNAIL expression in both DU-145 and PC-3 cell lines. [file 12943_2020_1267_MOESM6_ESM.tif]

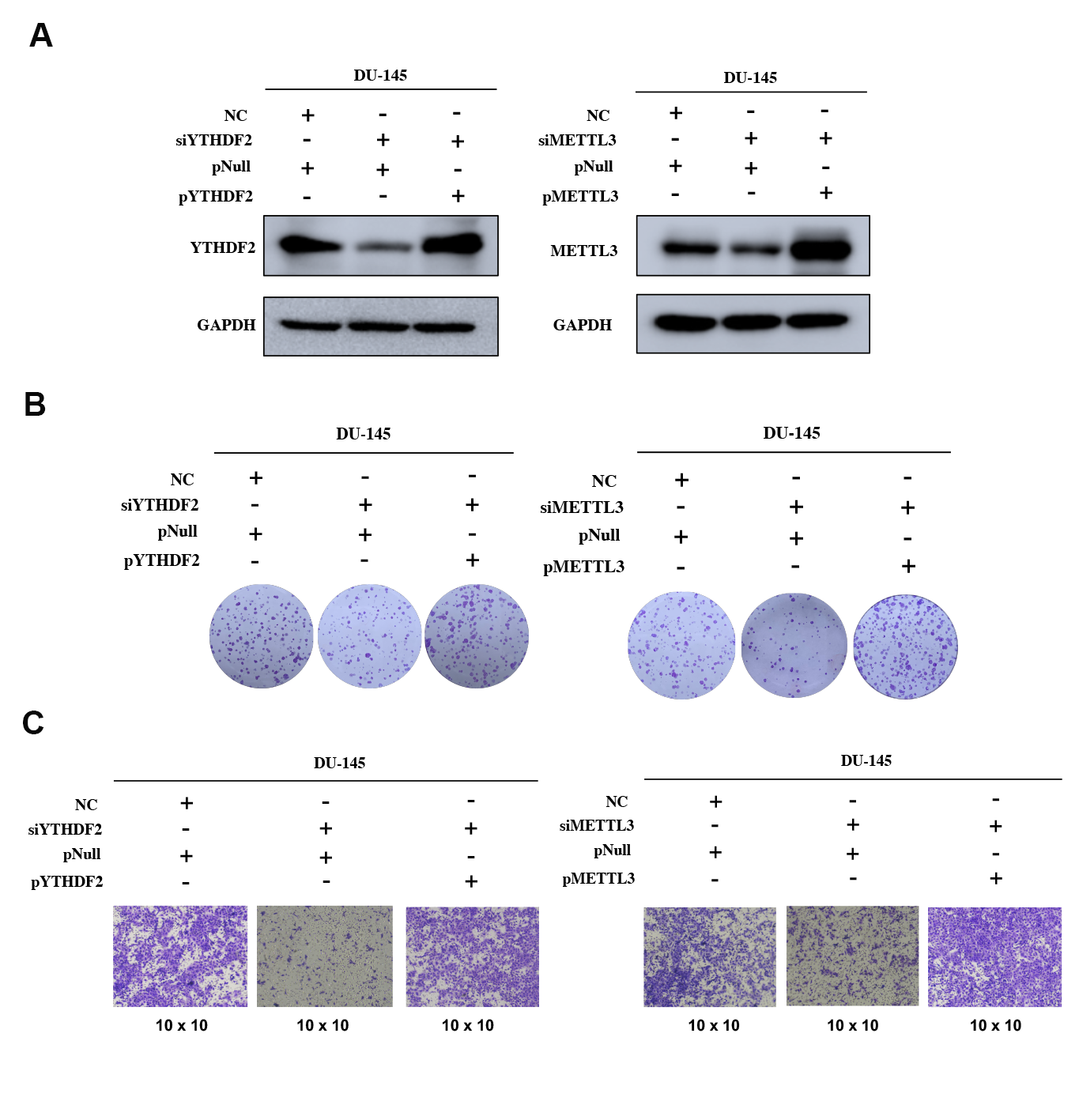

Supplement: Supplementary file 7 — Additional file 7: Figure S5. Overexpression of wild type YTDHF2 or METTL3 rescues the expression and cellular biofunction. (A) Western blot assay. The protein levels of YTHDF2 and METTL3 were rescued by wild type overexpression plasmid in DU-145 cell line. (B)-(C) Colony formation and trans-well assy. Cell colony ability and migration ability were rescued by wild type overexpression of YTHDF2 or METTL3 after knock-down in DU-145 cell line. [file 12943_2020_1267_MOESM7_ESM.tif]

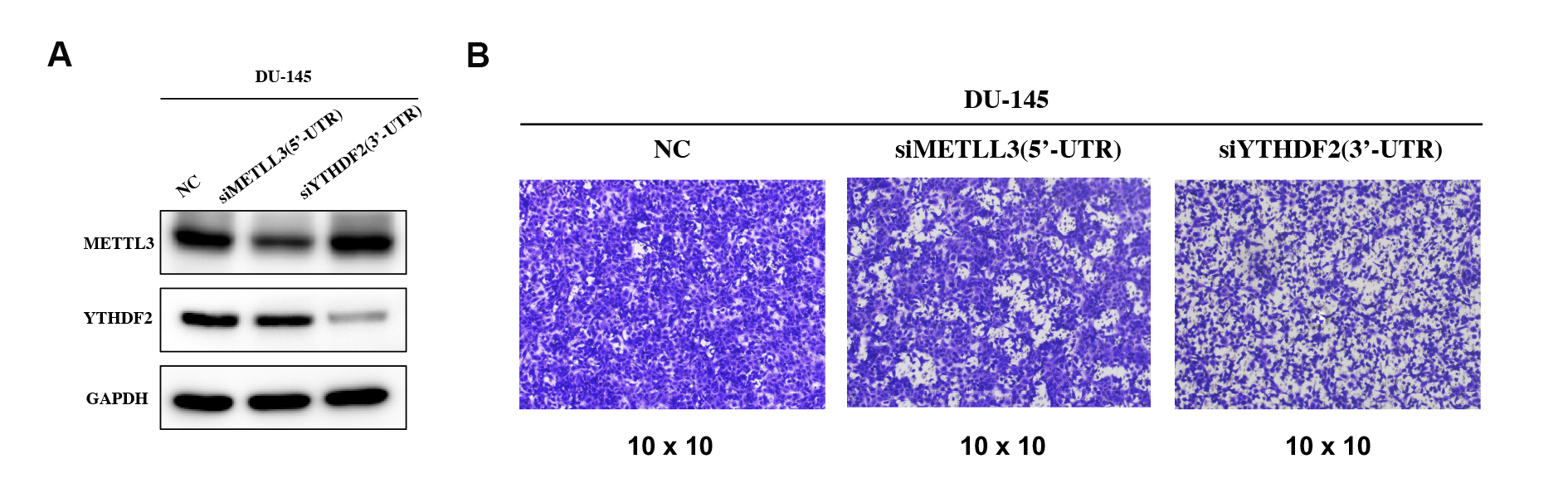

Supplement: Supplementary file 8 — Additional file 8: Figure S6. 5’UTR targeting siRNA of METTL3 and 3’UTR targeting siRNA of YTHDF2 consistently knock down endogenous expression and inhibited the cell migration. (A) Western blot assay. The knock-down effect of UTR targeting siRNAs. (B) Trans-well assay. Cell migration ability was inhibited after knock-down of YTHDF2 and METTL3 with UTR targeting siRNAs. [file 12943_2020_1267_MOESM8_ESM.tif]

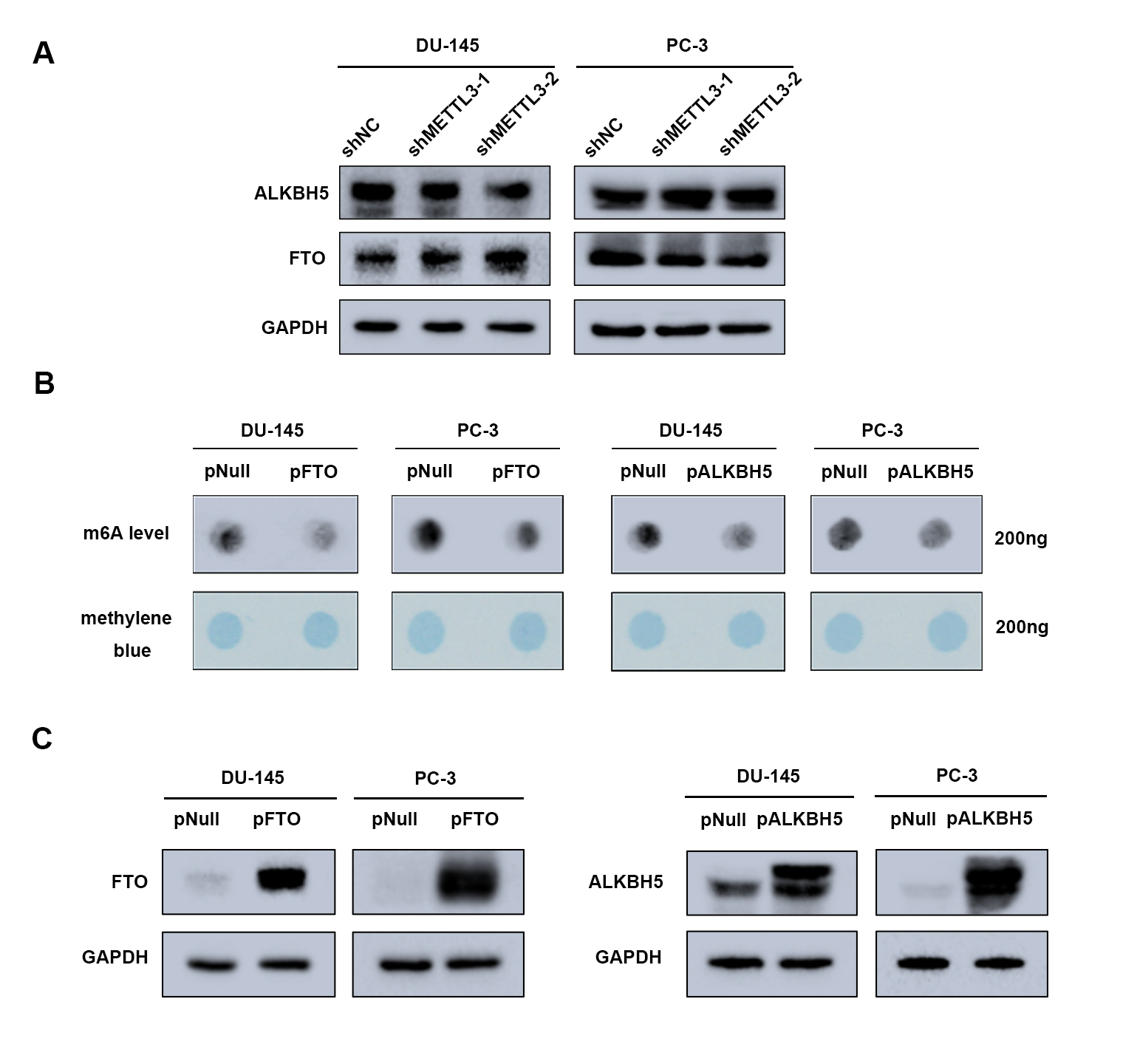

Supplement: Supplementary file 9 — Additional file 9: Figure S7. Knocking down METTL3 has no effect on the expression of FTO and ALKBH5. (A) Western blot assay. Knocking down METTL3 didn’t change the expression of FTO or ALKBH5 at protein levels. (B) RNA m6A Dot-blot assay. Overexpression of FTO or ALKBH5 induced a slight downregulation of m6A levels in PCa cell lines at 200 ng total RNA concentration. (C) Western blot assay. The overexpression efficiencies of FTO and ALKBH5 were identified in PCa cell lines. [file 12943_2020_1267_MOESM9_ESM.tif]

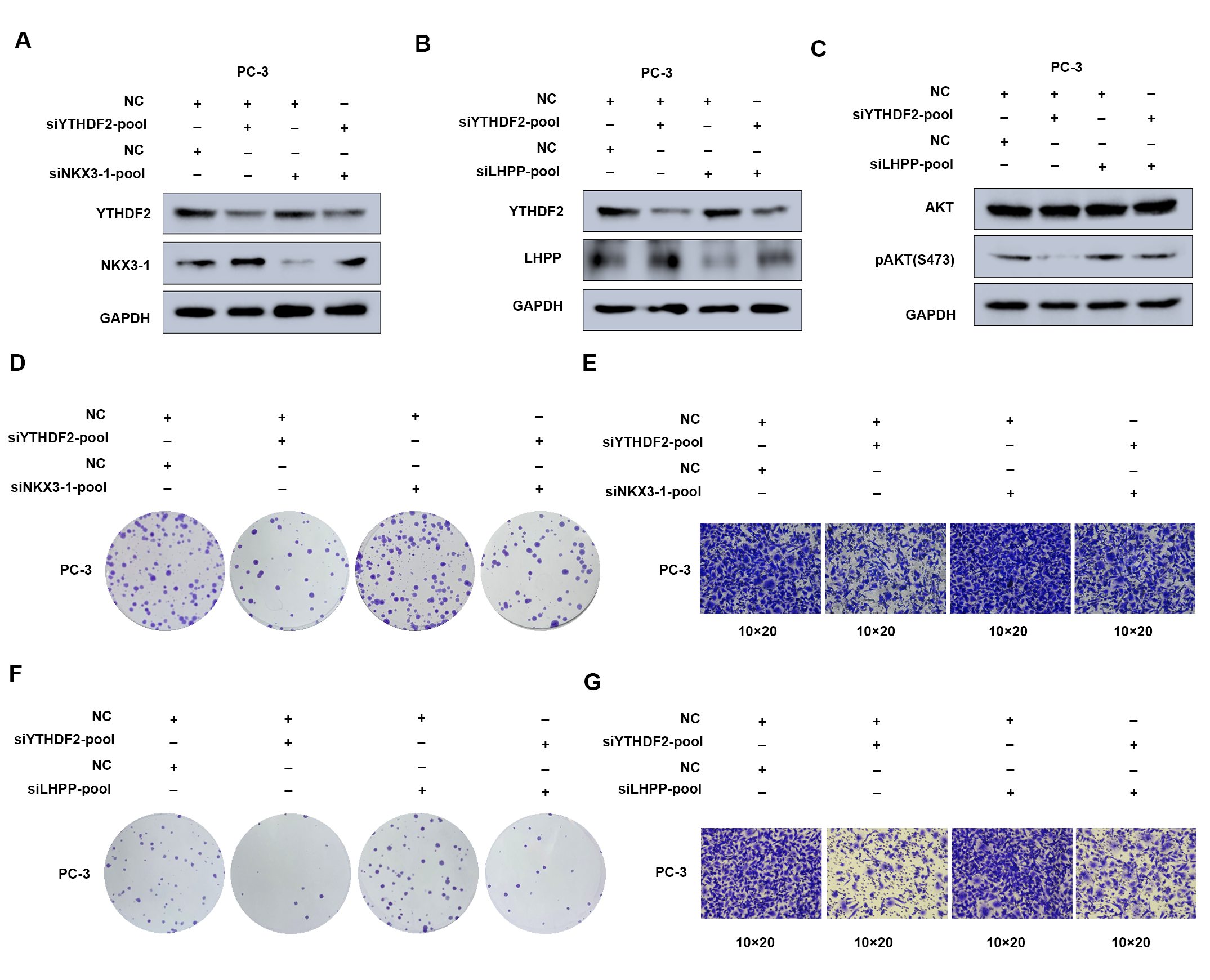

Supplement: Supplementary file 10 — Additional file 10: Figure S8. Rescue experiments between YTHDF2 and LHPP/NKX3–1/pAKT. (A)-(C) The protein level changes of rescue experiments in PC-3 cell line. (A) Western blot assay. The suppressed expression of NKX3–1 induced by siNKX3–1-pool was partially restored by knock-down of YTHDF2 (siYTHDF2-pool) at protein level. (B) Western blot assay. The suppressed expression of LHPP induced by siLHPP-pool was partially restored by knock-down of YTHDF2 (siYTHDF2-pool) at protein level (C) Western blot assay. Knock-down of LHPP partially rescued the expression of pAKT(s473) at protein level reduced by knock-down of YTHDF2. (D)-(G) The phenotypes induced by rescue experiments. (D) Colony formation assay (representative wells were presented). Colony formation rate promoted by siNKX3–1-pool was partially inhibited by knocking down YTHDF2. (E) Trans-well assay (representative wells were presented). The migration ability promoted by siNKX3–1-pool was partially inhibited by knocking down YTHDF2. The photograph was taken under 20× objective (10 × 20). (F) Colony formation assay (representative wells were presented). Colony formation rate promoted by siLHPP-pool was partially inhibited by knocking down YTHDF2. (G) Trans-well assay (representative wells were presented). The migration ability promoted by siLHPP-pool was partially inhibited by knocking down YTHDF2. The photograph was taken under 20× objective (10 × 20). [file 12943_2020_1267_MOESM10_ESM.tif]

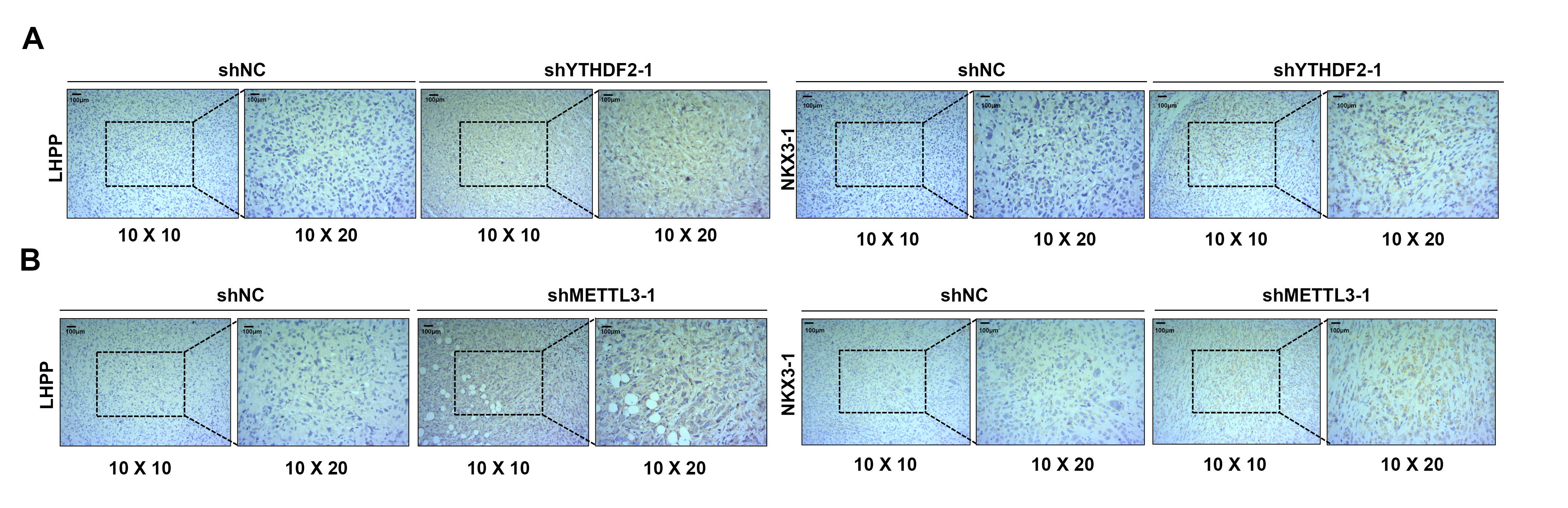

Supplement: Supplementary file 11 — Additional file 11: Figure S9. IHC staining of LHPP and NKX3–1 in subcutaneous xenografts after knocking down of YTHDF2 or METTL3. (A)-(B) Representative IHC staining micrographs of LHPP, NKX3–1 in tumor xenografts were conducted. Scale bar = 100 μm. [file 12943_2020_1267_MOESM11_ESM.tif]

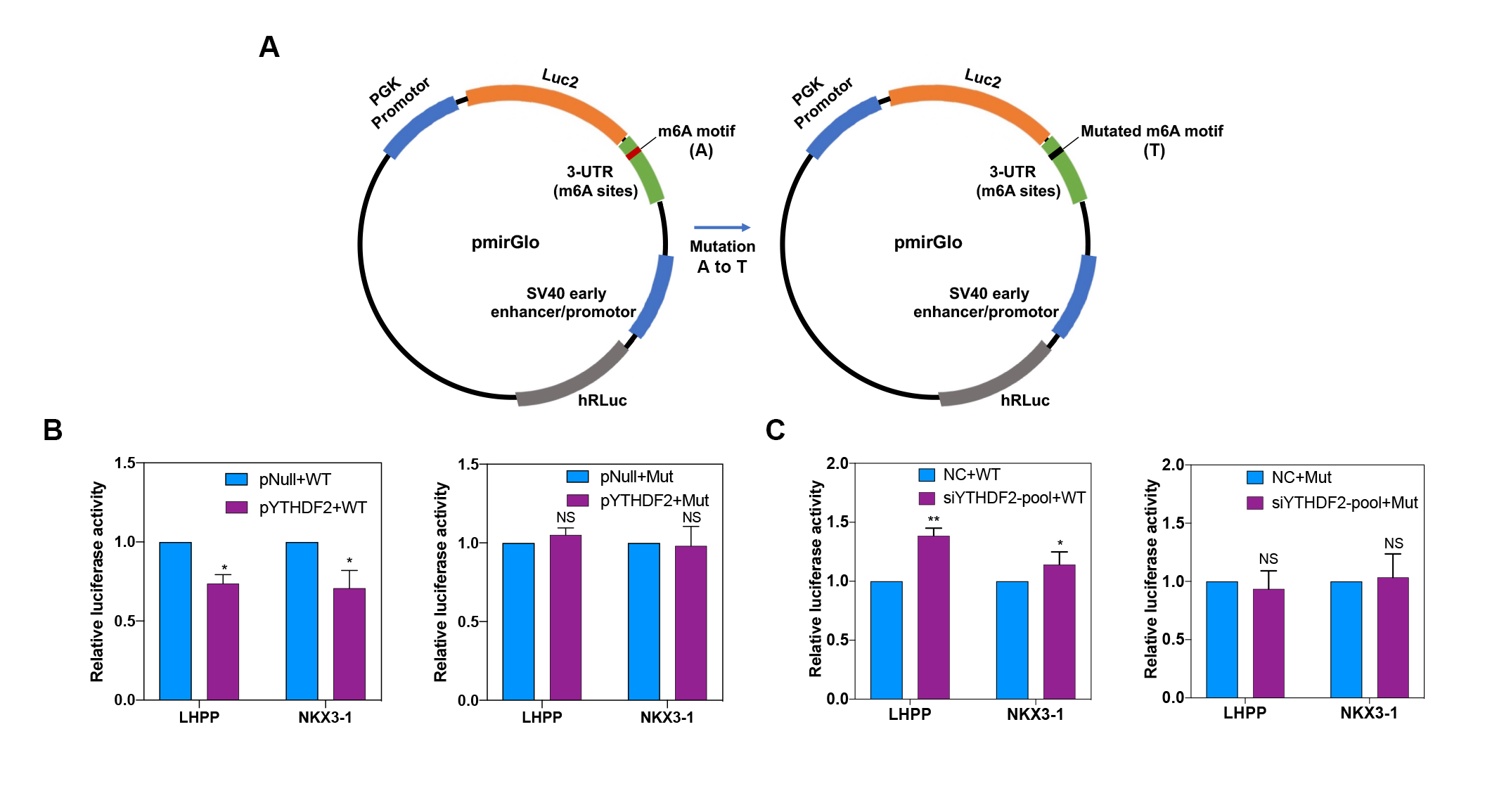

Supplement: Supplementary file 12 — Additional file 12: Figure S10. Mutated m6A sites inhibited the binding of YTHDF2 to LHPP and NKX3–1. (A) The schematic diagram presented the dual luciferase vector pmirGlo and inserted wildtype and mutated sequences (A to T). (B) Dual luciferase activity assay. Overexpression of YTHDF2 inhibited the luciferase activity of wild type LHPP or NKX3–1 but not of the mutated in PC-3 cell line. Student’s t-test was used for statistics analysis. (C) Knock-down of YTHDF2 (siYTHDF2-pool) consistently elevated the luciferase activity of wild type LHPP or NKX3–1 but not of the mutated in PC-3 cell line. Error bars represent the SD obtained from at least three independent experiments and student’s t-test was used for statistics analysis. *P ≤ 0.05, **P ≤ 0.01, ***P ≤ 0.001. [file 12943_2020_1267_MOESM12_ESM.tif]

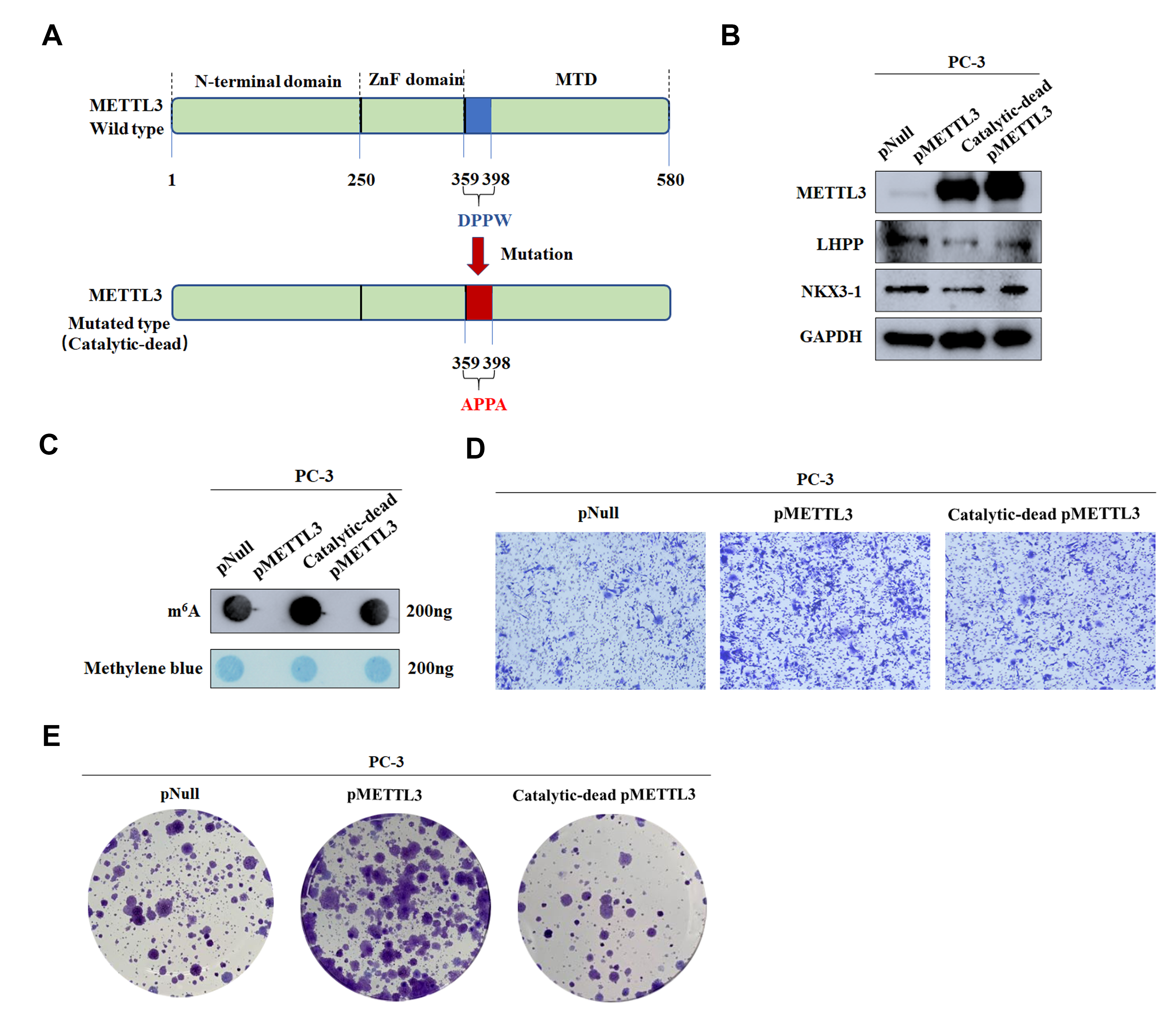

Supplement: Supplementary file 13 — Additional file 13: Figure S11. Catalytic dead METTL3 loses the function of m6A formation, targets inhibition and tumor progression promotion. (A) Schematic diagram. The catalytic region (359 to 398, DPPW) of wild type METTL3 was mutated into APPA. (B) Western blot assay. The expression of LHPP and NKX3–1 were downregulated after wild type transfection but not mutated type. (C) RNA m6A dot-blot assay. Overexpression of catalytic dead METTL3 couldn’t promote total m6A level like wild type METTL3. (D) and (E) Catalytic dead METTL3 has little effect on proliferation and migration. [file 12943_2020_1267_MOESM13_ESM.tif]

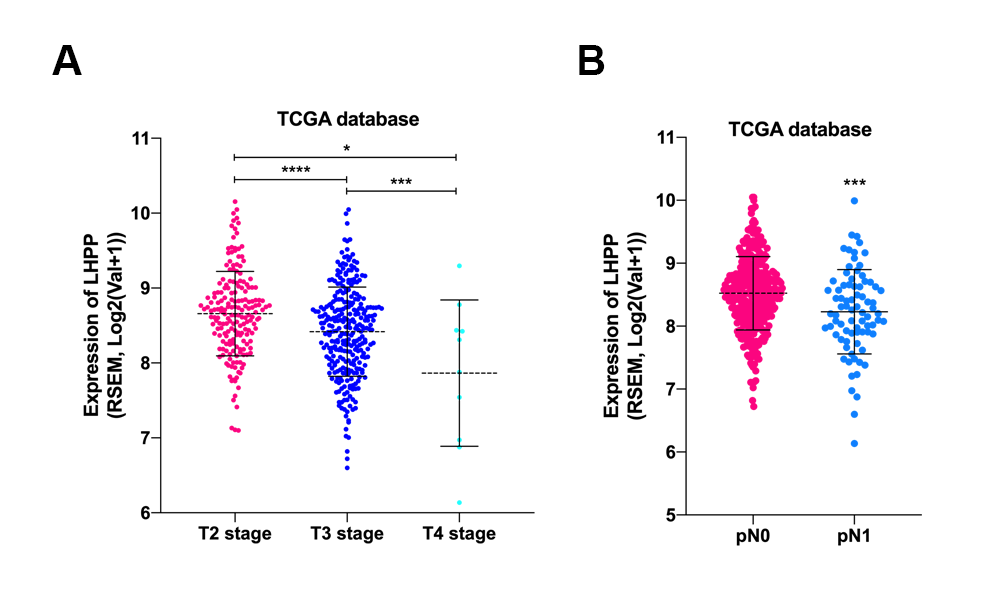

Supplement: Supplementary file 14 — Additional file 14: Figure S12. Subgroup expression pattern of LHPP in TCGA database. (A) Clinical stage analysis. LHPP had lower expression in upregulated stages. One-way ANOVA with multiple comparison test (Bonferoni correction) was used for statistics analysis. (B) Lymph node metastasis analysis. LHPP was downregulated in PCa tissues with lymph node metastasis (pN1) compared with non-lymph nodes metastasis (pN0). Student’s t-test was used for statistics analysis. *P ≤ 0.05, **P ≤ 0.01, ***P ≤ 0.001. [file 12943_2020_1267_MOESM14_ESM.tif]

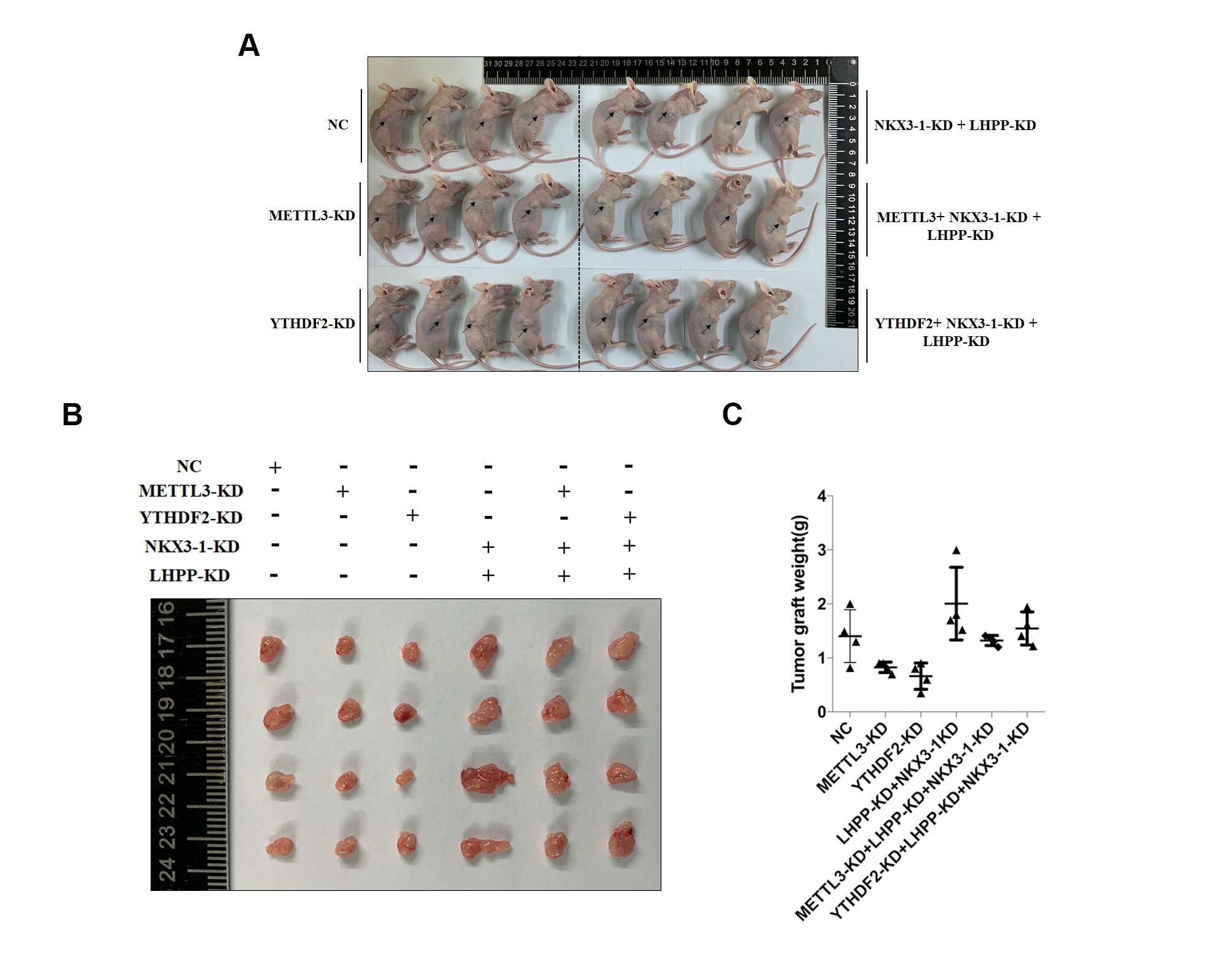

Supplement: Supplementary file 15 — Additional file 15: Figure S13. Knock down of YTHDF2 or METTL3 partially rescued the mice tumor growth induced by knock down of LHPP and NKX3–1. (A) The subcutaneous tumor models were observed in six different groups (blank arrows indicated tumor xenografts). (B) The BALB/c nude mice were sacrificed for the xenografts, and the size was measured by the beside ruler. (C) The tumor weight of each xenograft was measured and plotted. [file 12943_2020_1267_MOESM15_ESM.tif]

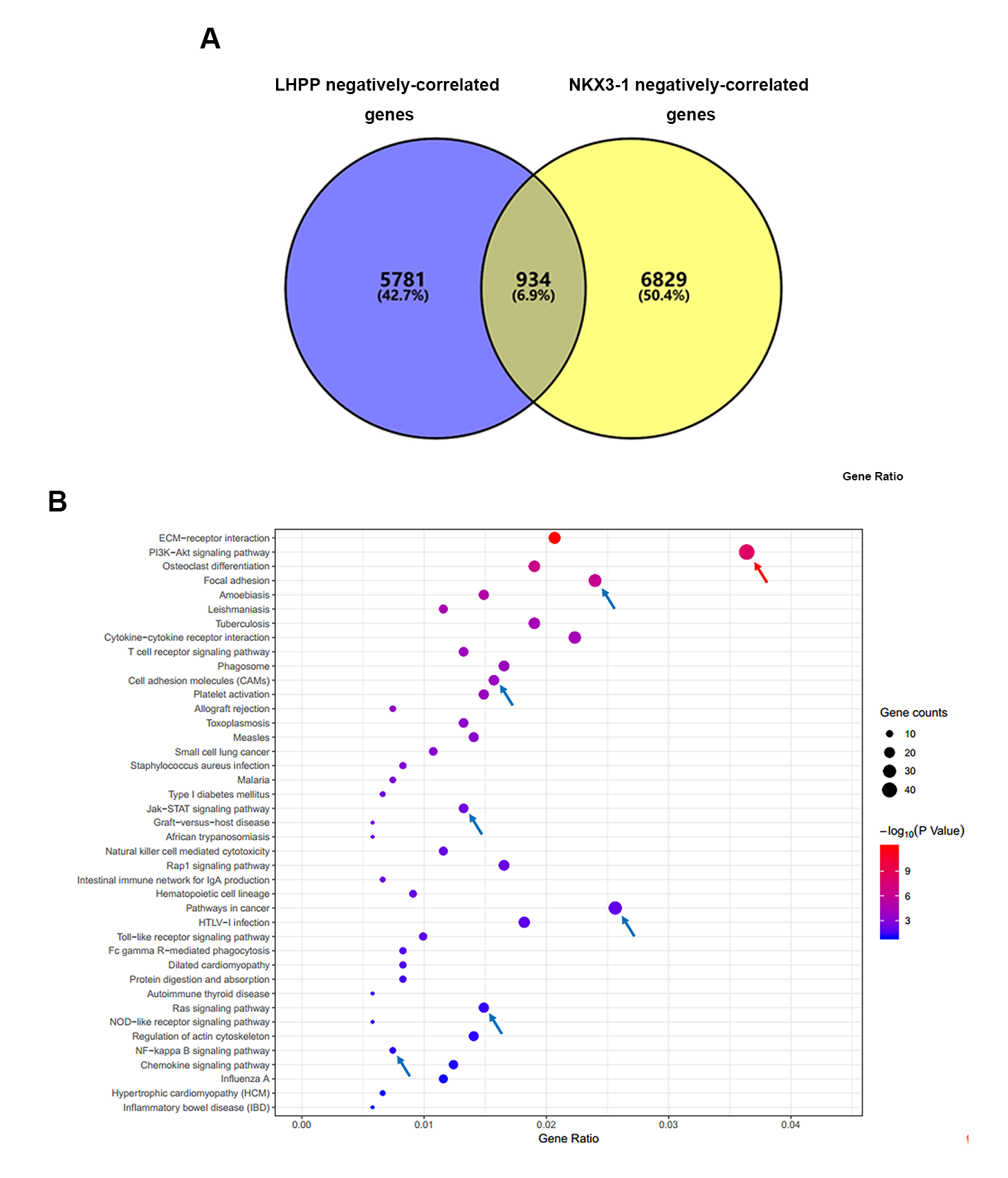

Supplement: Supplementary file 16 — Additional file 16: Figure S14. Multiple pathways associated with tumors are mainly involved in YTHDF2 mediated PCa progression. (A) The Venn diagram was used to present the common negatively-correlated genes (934) with LHPP and NKX3–1, which were downloaded from TCGA database and analyzed by LinkedOmics. (B) KEGG pathway analysis with the 934 common genes shown in (A) indicated that multiple pathways including ‘PI3K-AKT pathway’ and ‘JAK-STAT pathway’ etc. (blue arrow) were mainly involved in PCa tumor progression. [file 12943_2020_1267_MOESM16_ESM.tif]

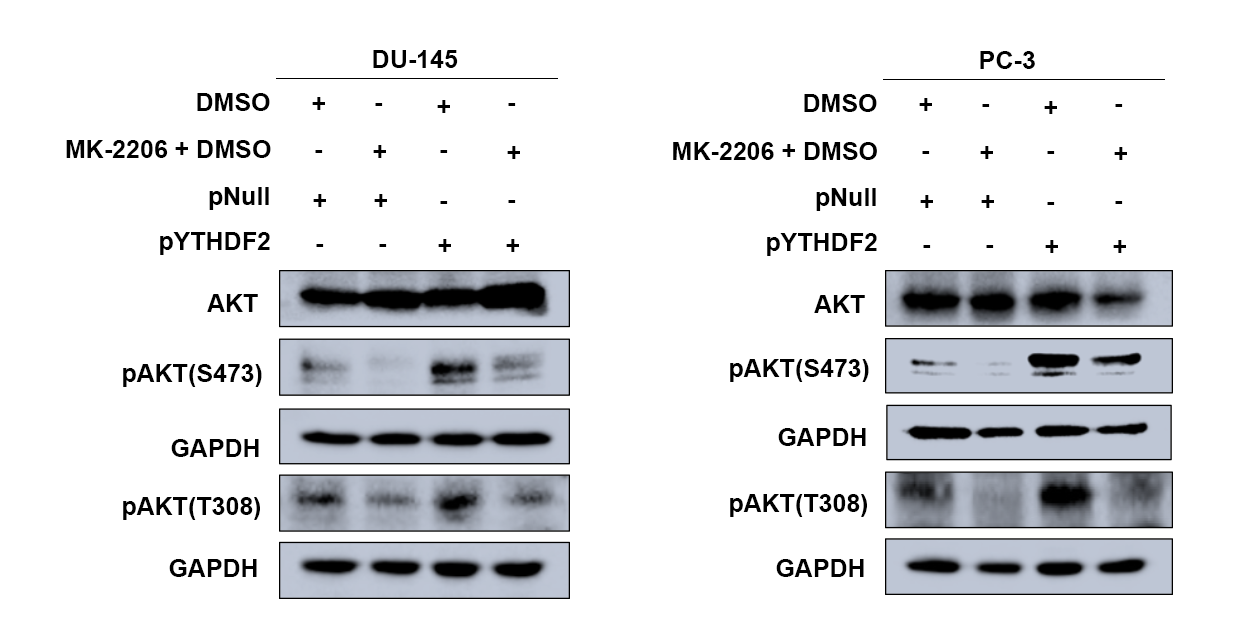

Supplement: Supplementary file 17 — Additional file 17: Figure S15. Overexpression of YTHDF2 could significantly rescued the reduced AKT phosphorylation level by AKT inhibitor. Western blot assay. Forced expression of YTHDF2 significantly rescued the inhibited pAKT(S473) and pAKT(T308) level by AKT inhibitor (MK-2206) in both DU-145 and PC-3 cell lines. [file 12943_2020_1267_MOESM17_ESM.tif]
